# Inequalities in treatment among patients with colon and rectal cancer: a multistate survival model using data from England national cancer registry 2012–2016

**DOI:** 10.1038/s41416-023-02440-6

**Published:** 2023-09-23

**Authors:** Suping Ling, Miguel-Angel Luque Fernandez, Manuela Quaresma, Aurelien Belot, Bernard Rachet

**Affiliations:** Inequalities in Cancer Outcome Network (ICON) group, Department of Non-communicable Disease Epidemiology, Faculty of Epidemiology and Population Health, London School of Hygiene & Tropical Medicine, Keppel Street, WC1E 7HT London, United Kingdom

**Keywords:** Health care, Cancer, Epidemiology

## Abstract

**Background:**

Individual and tumour factors only explain part of observed inequalities in colorectal cancer survival in England. This study aims to investigate inequalities in treatment in patients with colorectal cancer.

**Methods:**

All patients diagnosed with colorectal cancer in England between 2012 and 2016 were followed up from the date of diagnosis (state 1), to treatment (state 2), death (state 3) or censored at 1 year after the diagnosis. A multistate approach with flexible parametric model was used to investigate the effect of income deprivation on the probability of remaining alive and treated in colorectal cancer.

**Results:**

Compared to the least deprived quintile, the most deprived with stage I–IV colorectal cancer had a lower probability of being alive and treated at all the time during follow-up, and a higher probability of being untreated and of dying. The probability differences (most vs. least deprived) of being alive and treated at 6 months ranged between −2.4% (95% CI: −4.3, −1.1) and −7.4% (−9.4, −5.3) for colon; between −2.0% (−3.5, −0.4) and −6.2% (−8.9, −3.5) for rectal cancer.

**Conclusion:**

Persistent inequalities in treatment were observed in patients with colorectal cancer at every stage, due to delayed access to treatment and premature death.

## Introduction

Socioeconomic inequalities in colorectal cancer survival have been reported in England for many decades [1–6], with a deprivation gap (measured as the absolute difference in 1-year net survival between the most and least deprived quintile) ranging from −10.6% to −6.8% in 2006 [1], and there was no evidence of a reduction following the introduction of successive national cancer policies since 2000 [6]. Considerable efforts have been spent on identifying factors behind inequalities, of which most studies have focused on individual and tumour factors such as age, comorbidities and tumour stage (a proxy of late diagnosis). Yet, previous work in our research group has demonstrated that these factors explain only part of socioeconomic inequalities in cancer survival [4]. Besides, among patients who were recruited in a clinical trial and given equal treatments across socioeconomic status, the deprivation gap in colorectal cancer survival was much smaller than that in the general population [7]. These observations suggested that differential management and treatment of colorectal cancer may also contribute to such inequalities, despite that the National Health Service (NHS) in England is based on universal healthcare coverage. Although regional variations in treatment among colorectal cancer patients have been studied [8, 9], it remains largely unknown to what extent socioeconomic deprivation affects the probability and their timing of receiving treatments, accounting for the fact that some patients may not survive up to treatments.

Using data from National Cancer Registration and Analysis Service (NCRAS) in England between 2012 and 2016, this study aimed to investigate socioeconomic inequalities in access to treatment in patients with colon or rectal cancer at different tumour stages using a multistate modelling approach [10–12].

## Methods

### Patient and public involvement

Patients and members of the public were involved in prioritising the research questions, developing the application for funding, management of the research and will be involved in dissemination of research findings. In October 2021, April 2022, and February 2023, the planned research and relevant progress of Inequalities in Cancer Outcome Network (ICON) Programme was discussed with the ICON advisory group, comprising five people including one patient representative affected by cancer. Important contributions have related to refining or redefining our research questions to ensure that our research is relevant and translatable. Patient representatives will also help us to explain and present our research by contributing to lay summaries and to disseminate our findings by commenting on our visual outputs (such as infographics).

### Data sources and population

We used NCRAS to identify a cohort of patients diagnosed with colon and rectal cancer in England. NCRAS routinely collects clinical information on all cancer cases in England [13]. NCRAS are linked to systemic anti-cancer therapy (SACT) [14] and National Radiotherapy Dataset (RTDS) at patient- and tumour-level, and to Hospital Episodes Statistics Admitted Patient Care (HES APC) [15] databases at patient-level. Each patient is also linked to the ecological deprivation measure—Index of Multiple Deprivation (IMD) of the Lower-layer Super Output Areas (LSOA—population ranging from 1000 to 3000) of their residence at the time of their cancer diagnosis. We followed the Strengthening the Reporting of Observational Studies in Epidemiology (STROBE) Statement in reporting and conducting this study [16].

We followed data quality control processes for NCRAS described in Li et al. [17]. For the purpose of this study, inclusion and exclusion criteria are described as below. We included patients with a first primary colon (ICD-10 codes: C18) or rectal cancer (C19–C20), aged between 18 and 99 years at diagnosis between 1st Jan 2012 and 31st Dec 2016. The index date was the date of colon or rectal cancer diagnosis. In 295 (0.35%) colon and 11 (0.02%) rectal cancer patients having the same cancer record in HES APC within 120 days before the date of diagnosis in NCRAS, we treated these as the same diagnosis and used the earliest date of diagnosis across two databases as the index date (i.e. the date of cancer diagnosis). We excluded patients diagnosed via death certificate only, without the exact month and year of diagnosis, or with improper dates (i.e. death before diagnosis).

### Exposures and covariates

Each patient was allocated the income domain score of the IMD 2015 based on the proportion of people in receipt of means tested benefits in their LOSA [18]; this deprivation score was then categorised according to the quintiles of the national distribution of LSOAs. The quintiles of IMD 2015 income domain was used as the proxy of socioeconomic status as it is more comparable with measures of material deprivation [19]. The stage of cancer diagnosis (I, II, III, IV, and missing) reported by NCRAS was complemented through a pre-defined algorithm using clinical and pathological TNM staging information collected by NCRAS [20]. The presence of comorbidities, which may affect the treatment decision, including heart failure, myocardial infraction, chronic pulmonary disease, and diabetes with complications, were derived from HES APC [21]. Age at cancer diagnosis, sex, ethnicity and route to diagnosis were also extracted from NCRAS. The route to diagnosis was determined by the NCRAS team using multiple electronic health records datasets [22]; based on algorithms related to patient’s journey in the NHS during diagnostic periods, patients can be diagnosed via 2-week-wait route (whereby patients being urgently referred for suspected cancer by their GP can expect to be seen by a specialist within 2 weeks), screening, standard GP referral, emergency presentation, inpatient elective, and other outpatient.

### Outcomes

Outcomes included the date of any cancer treatment and the date of death within 1 year after diagnosis, in which death can occur before or after treatment. We chose to follow up patients for 1 year after diagnosis as treatment activities should be initiated within 1 year. Treatment could be colon or rectal resection (surgery), chemotherapy and radiotherapy. Surgery was ascertained by the presence of relevant OPCS-4 procedure codes in NCRAS and/or HES APC. If multiple procedures were undergone for the same patient, we used the earliest of the most extensive resection as the date of surgery. The use of chemotherapy was defined as the presence of anti-cancer regimens (excluding supportive regimens) in SACT or NCRAS, or relevant OPCS-4 codes for chemotherapy delivery in HES APC [23]. Similarly, the use of radiotherapy was determined by the record of radiotherapy in RTDS or NCRAS, or relevant OPCS-4 codes for radiotherapy delivery in HES APC.

### Statistical analysis

We described the characteristics at diagnosis of included patients with colon or rectal cancer by stage, with the median and interquartile range (IQR) for continuous and the number and proportion for categorical variables.

**Fig. 1:**
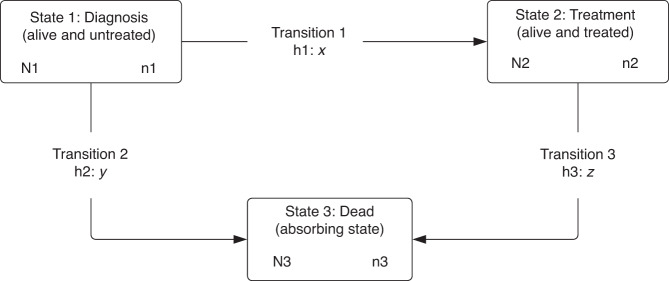
An overview of three states and three possible transitions. N1: The number of patients entering state 1; i.e., the total sample of each stage. n1: The number of patients staying at state 1 at the end of follow-up; i.e., those who did not die nor receive treatment. x: The number of patients moved from state 1 to state 2; i.e., those who received treatment. y: The number of patients moved from state 1 to state 3; i.e., those who died before receiving any treatment. N2: The number of patients entering state 2: i.e., those who received treatment (same as x). n2: The number of patients staying at state 2 at the end of follow-up; i.e., those who survived after receiving treatment. z: The number of patients moved from state 2 to state 3; i.e., those who died after receiving treatments. N3: The number of patients entering state 3; i.e., those who died during the follow-up, equal to the sum of y and z. n3: The number of patients staying at state 3 at the end of follow-up; as state 3 dead is an absorbing state, n3 is the same N3.

We used multistate models with three states: (1) diagnosis (alive and untreated), (2) treatment (alive and treated), and (3) death (i.e. the absorbing state), thus three transition intensities (h1: diagnosis to treatment, h2: diagnosis to death, and h3: treatment to death) to investigate the probability and the length of stay at each state [10]. Figure 1 illustrates three states and three possible transitions. All patients are followed-up from diagnosis to death or 365.24 days after diagnosis (at which time they were censored), and with an intermediate outcome, if present, the earliest date of receiving treatment. For those patients having a transition on the same day as the previous state (e.g. a patient was treated on the date of diagnosis), we manually added a partial day (a random number between 0.1 and 0.9) to their event time to include them in the analyses.

For each transition, we fitted a Royston-Parmar flexible parametric survival model using the survival time (days) to each outcome [24]. This multistate modelling approach allows accounting for the immortal time bias from patients who died before receiving any treatment. We assumed that the probability to move to the next state only depends on the present state (i.e. Markov assumption) [10]. The degree of freedom for the hazard function used in each model was determined by Akaike Information Criterion (AIC) and Bayesian Information Criterion (BIC) [25].

In all regression models, we included the main exposure i.e. income deprivation (5 quintiles from least to most deprived), and adjusted for age assuming a non-linear functional form (smooth function of age using restricted cubic splines with four knots placed at 5, 35, 65 and 95 percentiles), sex (men vs. women), ethnicity (White vs. Other), presence of heart failure, myocardial infarction, chronic pulmonary disease, and diabetes with complications (Yes vs. No), and route to diagnosis (emergency presentation, inpatient elective, other outpatient, screening, and 2-week-wait vs. standard GP referral). Methodological research on missing data is yet sparse in the context of multistate models, we therefore developed a complete-case analysis and only included those patients without missing data on covariates. Patients with missing stage (8.6% of colon and 6.6% of rectal cancer) were analysed separately. We also conducted sensitivity analyses by including more contemporary data—patients who were diagnosed between 2015 and 2016. We further stratified analyses by whether patients were diagnosed via screening, as screening is an unique diagnostic modality where patients did not seek medical attentions for symptoms. From the multistate models, we derived the probability and length of staying at each state by socioeconomic status assessing the differences between the most and least deprived cancer patients. We presented the results stratified by cancer sites (colon and rectum) and stages and reported all estimates with 95% confidence intervals (CIs). We conducted all analyses in Stata 16.1/MP (College Station, TX: StataCorp LLC). Clinical code lists and statistical codes used in the analyses are available at GitHub (https://github.com/supingling/colorectal_cancer).

## Results

### Cohort characteristics

The detailed flowchart of patients’ selection is shown in Supplemental Fig. S1. Of 85,137 and 48,798 patients with colon and rectal cancer, respectively, between 2012 and 2016, 1.5% and 0.9% did not meet inclusion criteria, 7.4% and 7.0% were excluded due to missing on deprivation, ethnicity and route to diagnosis, and further 7.9% and 6.0% patients were missing on stage, leaving 70,705 and 41,991 with stage I to IV colon and rectal cancer, respectively, included in our final analysis. Patients missing on stage only (6695 colon and 2950 rectal) were analysed separately. Missing data patterns are shown in Supplemental Table S1**:** the proportion of missing data is also higher in missing stages than others (nearly 20% in stage missing vs. <8% in other stages). The baseline characteristics of included and excluded patients are shown in Table S2.

The characteristics of included patients at cancer diagnosis, stratified by stage (I–IV), are shown in Table 1. Overall, the median age was 73.1 years (IQR: 64.4–80.6) for colon and 70.2 (IQR: 61.5–78.2) for rectal cancer. There were 32,751 (46.3%) and 15,187 (36.2%) women with colon and rectal cancer, respectively, and 95.1% of patients were White in both cancers. In total, 18.4% of colon cancer patients were diagnosed through emergency presentation but this figure was 7.0% for rectal cancer; The most common comorbidity was chronic pulmonary disease (>10%), followed by myocardial infarction and heart failure (<5%), and the least was diabetes with complications (<1%). Table S3 shows the characteristics of patients with missing stage of colon and rectal cancer. Compared to other stages, patients with missing stage, in both colon and rectal cancers, were considerably older, more deprived, more comorbid, and more likely to be diagnosed through emergency presentation.

**Table 1:** Baseline characteristics of included patients with colon (*N* = 70,705) or rectal (*N* = 41,991) cancer in England between 2012 and 2016.

*Colon cancer*	Total	Stage I	Stage II	Stage III	Stage IV
	*N* = 70,705	*N* = 11,832	*N* = 19,083	*N* = 21,354	*N* = 18,436
Age at diagnosis, years	73.1 (64.4–80.6)	71.2 (62.9–78.7)	74.2 (66.1–81.3)	72.7 (64.2–80.4)	73.2 (63.8–81.4)
Age group, years					
18-44	2727 (3.9%)	734 (6.2%)	625 (3.3%)	707 (3.3%)	661 (3.6%)
45-54	4333 (6.1%)	615 (5.2%)	977 (5.1%)	1398 (6.5%)	1343 (7.3%)
55-64	11,741 (16.6%)	2198 (18.6%)	2709 (14.2%)	3744 (17.5%)	3090 (16.8%)
65-74	21,435 (30.3%)	4076 (34.4%)	5739 (30.1%)	6512 (30.5%)	5108 (27.7%)
74-84	21,875 (30.9%)	3198 (27.0%)	6522 (34.2%)	6549 (30.7%)	5606 (30.4%)
≥85	8594 (12.2%)	1011 (8.5%)	2511 (13.2%)	2444 (11.4%)	2628 (14.3%)
Sex					
Men	37,954 (53.7%)	6662 (56.3%)	10,148 (53.2%)	11,242 (52.6%)	9902 (53.7%)
Women	32,751 (46.3%)	5170 (43.7%)	8935 (46.8%)	10,112 (47.4%)	8534 (46.3%)
Ethnicity					
White	67,221 (95.1%)	11,258 (95.1%)	18,258 (95.7%)	20,219 (94.7%)	17,486 (94.8%)
Other ethnicities	3484 (4.9%)	574 (4.9%)	825 (4.3%)	1135 (5.3%)	950 (5.2%)
Income 2015 quintile					
1 – Least deprived	15,612 (22.1%)	2655 (22.4%)	4211 (22.1%)	4840 (22.7%)	3906 (21.2%)
2	16,374 (23.2%)	2769 (23.4%)	4505 (23.6%)	5005 (23.4%)	4095 (22.2%)
3	14,919 (21.1%)	2454 (20.7%)	4098 (21.5%)	4435 (20.8%)	3932 (21.3%)
4	12,946 (18.3%)	2144 (18.1%)	3498 (18.3%)	3861 (18.1%)	3443 (18.7%)
5 – Most deprived	10,854 (15.4%)	1810 (15.3%)	2771 (14.5%)	3213 (15.0%)	3060 (16.6%)
Comorbidity					
Heart failure	2248 (3.2%)	367 (3.1%)	635 (3.3%)	608 (2.8%)	638 (3.5%)
Myocardial infarction	2966 (4.2%)	488 (4.1%)	891 (4.7%)	857 (4.0%)	730 (4.0%)
Diabetes with complications	529 (0.7%)	94 (0.8%)	141 (0.7%)	139 (0.7%)	155 (0.8%)
Chronic pulmonary disease	8935 (12.6%)	1635 (13.8%)	2488 (13.0%)	2490 (11.7%)	2322 (12.6%)
Route to diagnosis					
Emergency presentation	12,980 (18.4%)	1227 (10.4%)	3225 (16.9%)	3337 (15.6%)	5191 (28.2%)
GP referral	17,798 (25.2%)	3393 (28.7%)	4618 (24.2%)	5370 (25.1%)	4417 (24.0%)
Inpatient elective	2521 (3.6%)	492 (4.2%)	667 (3.5%)	802 (3.8%)	560 (3.0%)
Other outpatient	5221 (7.4%)	959 (8.1%)	1512 (7.9%)	1409 (6.6%)	1,341 (7.3%)
Screening	8754 (12.4%)	2870 (24.3%)	2357 (12.4%)	2762 (12.9%)	765 (4.1%)
TWW	23,431 (33.1%)	2891 (24.4%)	6704 (35.1%)	7674 (35.9%)	6162 (33.4%)

*Rectal cancer*	Total	Stage I	Stage II	Stage III	Stage IV
	*N* = 41,991	*N* = 9510	*N* = 7504	*N* = 16,346	*N* = 8631
Age at diagnosis, years	70.2 (61.5–78.2)	70.6 (63.0–78.2)	72.6 (63.7–80.1)	68.7 (60.2–77.0)	70.4 (60.6–78.8)
Age group, years					
18–44	1401 (3.3%)	232 (2.4%)	184 (2.5%)	646 (4.0%)	339 (3.9%)
45–54	3723 (8.9%)	675 (7.1%)	521 (6.9%)	1677 (10.3%)	850 (9.8%)
55–64	9076 (21.6%)	1969 (20.7%)	1392 (18.6%)	3890 (23.8%)	1825 (21.1%)
65–74	13,217 (31.5%)	3398 (35.7%)	2237 (29.8%)	5113 (31.3%)	2469 (28.6%)
74–84	11,067 (26.4%)	2460 (25.9%)	2341 (31.2%)	3982 (24.4%)	2284 (26.5%)
≥85	3507 (8.4%)	776 (8.2%)	829 (11.0%)	1038 (6.4%)	864 (10.0%)
Sex					
Male	26,804 (63.8%)	5844 (61.5%)	4815 (64.2%)	10,579 (64.7%)	5566 (64.5%)
Female	15,187 (36.2%)	3666 (38.5%)	2689 (35.8%)	5767 (35.3%)	3065 (35.5%)
Ethnicity					
White	39,935 (95.1%)	9075 (95.4%)	7143 (95.2%)	15,522 (95.0%)	8195 (94.9%)
Other ethnicities	2056 (4.9%)	435 (4.6%)	361 (4.8%)	824 (5.0%)	436 (5.1%)
Income 2015 quintile					
1 – Least deprived	8815 (21.0%)	2138 (22.5%)	1614 (21.5%)	3389 (20.7%)	1674 (19.4%)
2	9488 (22.6%)	2248 (23.6%)	1705 (22.7%)	3705 (22.7%)	1830 (21.2%)
3	8965 (21.3%)	2046 (21.5%)	1564 (20.8%)	3520 (21.5%)	1835 (21.3%)
4	7865 (18.7%)	1685 (17.7%)	1425 (19.0%)	3012 (18.4%)	1743 (20.2%)
5 – Most deprived	6858 (16.3%)	1393 (14.6%)	1196 (15.9%)	2720 (16.6%)	1549 (17.9%)
Comorbidity					
Heart failure	859 (2.0%)	239 (2.5%)	176 (2.3%)	245 (1.5%)	199 (2.3%)
Myocardial infarction	1349 (3.2%)	375 (3.9%)	269 (3.6%)	435 (2.7%)	270 (3.1%)
Diabetes with complications	259 (0.6%)	67 (0.7%)	50 (0.7%)	91 (0.6%)	51 (0.6%)
Chronic pulmonary disease	4292 (10.2%)	1141 (12.0%)	820 (10.9%)	1415 (8.7%)	916 (10.6%)
Route to diagnosis					
Emergency presentation	2920 (7.0%)	356 (3.7%)	522 (7.0%)	791 (4.8%)	1251 (14.5%)
GP referral	11,226 (26.7%)	2969 (31.2%)	1935 (25.8%)	4193 (25.7%)	2129 (24.7%)
Inpatient elective	1557 (3.7%)	400 (4.2%)	261 (3.5%)	599 (3.7%)	297 (3.4%)
Other outpatient	2281 (5.4%)	730 (7.7%)	435 (5.8%)	687 (4.2%)	429 (5.0%)
Screening	5215 (12.4%)	1997 (21.0%)	858 (11.4%)	1927 (11.8%)	433 (5.0%)
TWW	18,792 (44.8%)	3058 (32.2%)	3493 (46.5%)	8149 (49.9%)	4092 (47.4%)

TWW: 2-week-wait referral; comorbidity: patients can have more than one condition listed.

### Descriptions of three transitions and models

Figure 1 shows an overview of three states and transitions and Fig. S2 presents the number of patients entering and staying at each state (i.e. diagnosis: alive and untreated, treatment: alive and treated, and dead) and experiencing each transition (h1: diagnosis to treatment, h2: diagnosis to death, and h3: treatment to death) by cancer and stage. In stage I and II, compared to patients who died after treatment (h3), both colon and rectal patients who died before the treatment (h2) were older (e.g. stage I colon cancer, median age 85.6 vs. 78.3 years old) and more comorbid than those who died after treatment (h3). More patients at advanced stages, compared with early stages, died before receiving any treatment, in both colon (33.7% of stage IV vs. 2.0% stage I) and rectal (21.3% stage IV vs. 1.3% stage I) cancer.

The degree of freedom selection for each Royston-Parmar Flexible Parametric survival model by cancer and stage are shown in Table S4. Transition-specific Hazard ratios (HRs) for socioeconomic status are shown in Fig. S3 and Table S5. For both cancers, compared with the least deprived quintile, other patients had a decreased risk of receiving treatment in all stages, with a larger effect size in patients with stage IV cancers and a gradient across quintiles of income deprivation. We also observed an increased risk of death before and after receiving treatment, with a larger effect size for death after than before treatment for most stages (Fig. S3; Table S5).

### Probability of staying at each state

**Fig. 2:**
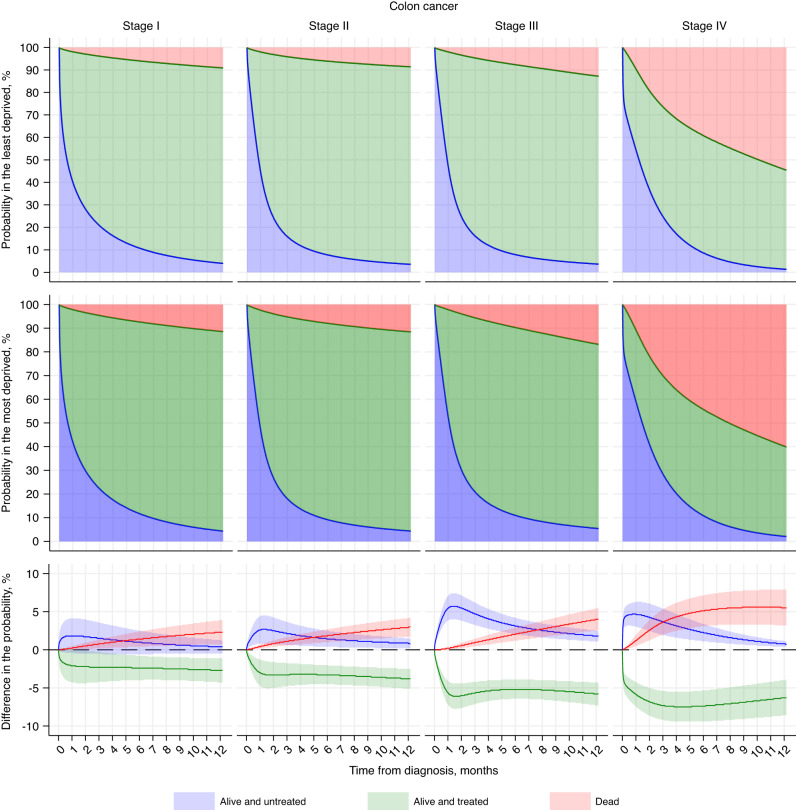
Probability of at each state in the least and most deprived and their differences in patients with stage I–IV colon cancer in England between 2012 and 2016 (age of 75). Three colours represent three states (blue: alive and untreated; green: alive and treated; red: dead). The probability of staying at each state by time since diagnosis (months) are shown for a white, male, 75-year-old patient without comorbidity, and with standard GP referral route who was in the least deprived quintile (first row) and in the most deprived quintile (second row), and differences between them (most vs. least deprived) in the probability (third row).

**Fig. 3:**
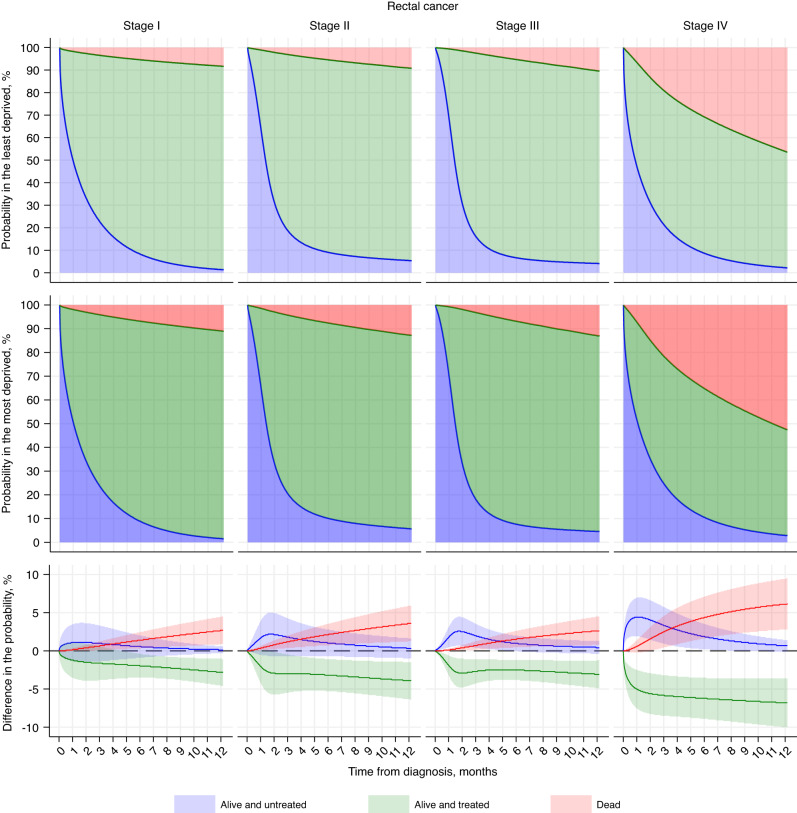
Probability of at each state in the least and most deprived and their differences in patients with stage I–IV rectal cancer in England between 2012 and 2016 (age of 75). Three colours represent three states (blue: alive and untreated; green: alive and treated; red: dead). The probability of staying at each state by time since diagnosis (months) are shown for a white, male, 75-year-old patient without comorbidity, and with standard GP referral route who was in the least deprived quintile (first row) and in the most deprived quintile (second row), and differences between them (most vs. least deprived) in the probability (third row).

Probabilities and differences (most vs. least deprived) in probability of staying alive and untreated, alive and treated, or dead, by months since diagnosis are shown in Fig. 2 for stage I to IV colon cancer and Fig. 3 for rectal cancer. These estimates were reported for the least and most deprived 75-year-old patients and all other covariates were set as reference group (i.e. male, White ethnicity, without any of these four comorbidities, and standard GP referral). Overall, we observed consistent deprivation gaps (i.e. the absolute difference in the probability comparing the most deprived to the least deprived) in treatment and death across cancer sites and stages. Compared to the least deprived, 75-year-old deprived patients with colon or rectal cancer, had a lower probability of receiving treatment, and a higher probability of staying untreated and of dying (Figs. 2 and 3).

In stage I– IV of colon cancer, the deprivation gap in remaining alive and treated dramatically increased within 1 month after diagnosis and stabilised thereafter. The gap at 6 months widened steadily with increasing stage from −2.4% (95% CI: −4.0, −0.8) in stage I to −7.4% (95% CI: −9.4, −5.3) for stage IV (Fig. 2; Table S6). A similar pattern though less clear was observed in remaining alive and untreated at 1 month after diagnosis, with a gap below 3% in stage I and II and around 5% in stage III and IV, but this gap narrowed towards null at 1 year. The deprivation gap in death was, however, progressively increasing during the whole study period, to 2.3% (95% CI: 0.7, 3.9) for stage I and up to 5.5% (95% CI: 3.2, 7.9) for stage IV at 1 year (Fig. 2; Table S6).

Comparable patterns were observed for rectal cancer stage I–IV, with smaller deprivation gaps in the probability of remaining alive and untreated but similar in the other two states (Fig. 3; Table S6). Differences (most vs. least deprived) in the probability of being alive and untreated at 1 month ranged between 1.1% (95% CI: −1.3, 3.5) in stage I and 4.4% (95% CI: 1.9, 6.9) in stage IV; of remaining alive and treated at 6 months between −2.0% (95% CI: −3.5, −0.4) and −6.2% (95% CI: −8.9, −3.5); and of death at 1 year between 2.7% (95% CI: 0.9, 4.5) and 6.1% (95% CI: 2.8, 9.4).

### Length of stay at each state

**Fig. 4:**
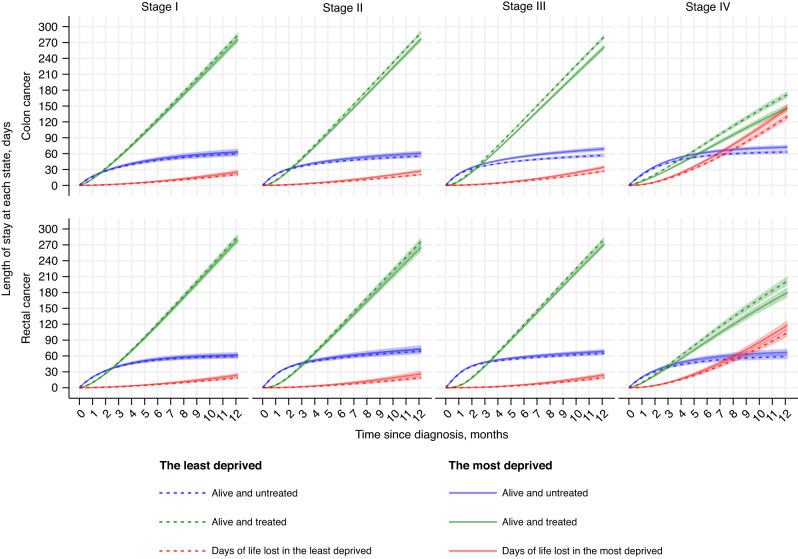
Length of stay at each state in the least and most deprived quintiles in patients with stage I–IV colon and rectal cancer in England between 2012 and 2016. Three colours represent three states (blue: alive and untreated; green: alive and treated; red: dead). The length of staying at each state (days) by time since diagnosis (months) are shown for a white, male, 75-year-old patient without comorbidity, and with standard GP referral route who was in the least deprived quintile (dash line) and in the most deprived quintile (solid line) and in colon (top panel) and rectal (bottom panel) cancer.

Figure 4 shows the length of stay at alive and untreated, alive and treated, and dead (days of life lost) in the least and most deprived patients with stage I–IV colon and rectal cancer. Consistent with estimates of probabilities, the most deprived patients spent less days being alive and treated, but more days being alive and untreated (waiting for the treatment), or had more days of life lost (died earlier), indicating a later enter to and an earlier exit from “treatment” state than the least deprived quintile. Differences between the most and least deprived quintiles increased over time since diagnosis in all tumour stages of both cancers, and were larger in colon than rectal cancer, and in more advanced than early stages (Fig. 4; Table S7).

Of 360 days after diagnosis, the most deprived patients with stage I colon cancer, compared to the least deprived, typically spent 8.5 days less (95% CI: −14.2, −2.7) being alive and treated, of which 3.8 days (95% CI: −1.8, 9.3) were due to the difference in length of stay at alive and untreated (delayed treatment), and 4.7 more days (95% CI: 1.4, 8.0) of life were lost. The difference in being alive and treated increased along with the stage; in stage IV, the most deprived spent 24.6 days less (95% CI: −31.4,−17.8) in this state (Fig. 4; Table S7). In early stages, differences in alive and treated were related to similar number days of delayed treatment and days of life lost (3.8 delays vs. 4.7 days lost in stage I; 5.6 vs. 6.3 days in stage II), but more days of delays than lost in stage III (12.0 vs, 7.2 days), and vice versa in stage IV (9.0 vs. 15.6 days).

In rectal cancer, the differences in days staying at alive and treated were −7.0 (95% CI: −12.7, −1.4) in stage I, −11.1 days (95% CI: −18.6, −3.6) in stage II, −9.1 days (95% CI: −14.0, −4.3) in stage III, and −21.7 days (95% CI: −31.0, −12.4) in stage IV (Table S7) at 1 year after diagnosis. In contrast to colon cancer, more days of such deprivation gaps were due to premature death than delayed treatment at all stages (Fig. 4; Table S7).

### Sensitivity analyses

Figures S4, S5 and Table S6, S7 show results of patients with missing stage colon and rectal cancer between 2012 and 2016 in England. In patients with missing stage colon cancer, differences between the most and least deprived quintiles in probability and length of stay at three states were similar to those patients with stage IV, but with larger uncertainties due to a smaller sample size, except that the most deprived patients with missing stage rectal cancer had a higher probability of death (5.1%; 95% CI: 0.1, 10.1), and a lower probability of being alive, regardless of treated or untreated.

Sensitivity analyses by including patients diagnosed only between 2015 and 2016 are shown in Fig. S6 (probabilities in colon cancer), Fig. S7 (probabilities in rectal cancer), and Fig. S8 (length of stay in colon and rectal cancer). These estimates were indistinguishable with that of the main analyses, except with larger uncertainties due to a smaller sample size in both cancers. Stratified analyses by whether patients were diagnosed via screening are shown in Fig. S9, Fig. S10, and Fig. S11. Both stage I-III colon and rectal patients diagnosed via screening had much lower probability of death and higher probability of treatment than those diagnosed via other routes, and we found no clear evidence of inequalities in these screen-detected patients. As stage IV patients were rarely diagnosed via screening, inequalities in this subgroup was inconclusive due to the small sample size.

## Discussion

Using data from 70,705 and 41,991 patients diagnosed with stage I–IV colon and rectal cancer in England between 2012 and 2016, we found persistent socioeconomic inequalities in access to treatment and premature death in every stage of colon and rectal cancer after controlling for age at cancer diagnosis, sex, ethnicity, route to diagnosis and four major comorbidities. Compared to the least deprived quintile, the most deprived had a lower probability of staying alive and treated, and a higher probability of death during the year after diagnosis. These inequalities were greater in advanced than early stages during the whole study period. The most deprived also had a higher probability of being alive and untreated within 1 month after diagnosis, but such disparities narrowed towards null along with the follow-up time.

These estimates translated into a smaller number of days remaining alive and treated in the most than the least deprived (e.g. at 1 year after diagnosis, 169.3 vs. 144.7 days, i.e. 24.6 days less, in stage IV colon cancer), and more days being alive and untreated (e.g. 9.0 days more in stage IV colon cancer) as well as an earlier death (15.6 days earlier in stage IV colon cancer). Taken together, our findings indicate that, the deprivation gaps in treatment (i.e. being alive and treated) was due to both delayed access to treatment right after diagnosis (later enter to the “treatment” state) and premature death (days of life lost, earlier enter to the “death” state). We also observed a gradient across quintiles of income deprivation for being alive and treatment in both cancers at all stages (not shown).

This study has some strengths and limitations. We included a large sample of patients with colorectal cancer from the latest available data from cancer registries in England—a high-quality database with as high as 99% national coverage on cancer patients [13]. We also linked to hospital admission data, systemic treatment records, and radiotherapy data to capture complete treatment records for these patients [8, 14, 15]. It should be noted that some of these data were not collected for the research purpose and activities outside NHS were not recorded (~1%); as some variables (e.g. treatment) used in our analysis relied on clinical coding in electronic health records, we could not rule out misclassification. The multistate approach allows better description of the outcomes appearing over time (such as treatment) while tackling the potential issue of competing risks and immortal time bias [10], and using flexible parametric models allows capturing a variety of complex hazard functions [24]. Regarding the missing data ( < 10%), we could only conduct complete-case analysis (83% and 86% of all cases, respectively) due to the lack of methodological research for missing data in multistate modelling. However, both sensitivity analyses (analysis restricted to patients with missing stages, and analyses on patients diagnosed between 2015 and 2016) did not alter our main conclusions. Results on missing stages were mostly consistent with those of stage IV, and the second sensitivity analyses provided consistent results while using a more contemporary population (2015–16). Lastly, we used small area-based income to determine socioeconomic status, which may not fully reflect the individual’s income [26].

Many previous epidemiological research and literature reviews have reported less favourable results on receiving treatment for deprived patients with colon and/or rectal cancer [27–31], though different data sources, definitions of exposures and outcomes, or statistical methods were used. Of note, many previous studies have analysed non-stage-specific populations or even combined colorectal cancer patients [27–29, 32, 33], while our analyses were stratified by cancer sites and stages and there were large sample sizes in each subgroup. Different proportions of patients with two cancers and/or different stages in previous studies may affect their observed inequalities. Indeed, in our study, we observed larger socioeconomic gaps in colon than rectal cancer and in advanced than early stages, possibly due to more complex treatment strategies and higher risk of death in advanced stages of colorectal cancer. Adjustments for age and stage (and sites if applicable) in previous studies were useful [29, 34], but we stratified by sites and stages and also adjusted for other important confounders such as sex and ethnicity, and other clinical factors (route to diagnosis and comorbidities).

However, several studies from Europe, England and Scotland suggested no evidence of treatment delay associated with deprivation [32, 33, 35], or even showed that deprived patients actually received quicker treatment [34]. The key explanation for these findings is that deprived patients may be more likely to be diagnosed via emergency presentation route [36], which leads to immediate treatment intervention [29, 35]. Previous studies suggested that whether patient met cancer waiting time targets for treatment (i.e. no more than 62 days from the urgent referral to the start of treatment; no more than 31 days between a decision to treat and the start of treatment) does not affect their survival—“waiting time paradox” [37], as sicker patients would be seen and treated more quickly and nevertheless had worse outcomes. However, our current analyses showed that, under similar demographic, clinical and tumour conditions, the deprived patients were treated later and died earlier than the affluent, except when stage I-III colorectal patients were diagnosed via screening, among whom we found no evidence of socioeconomic inequalities. Further, time to treatment was measured from cancer diagnosis (usually pathological diagnosis in NCRAS) to the initiation of the treatment in our study, while several studies used time from first symptoms (or contact/consultation/referral) to treatment, in which the time interval between first symptoms to confirmed diagnosis should reflect delays in diagnosis rather than treatment [33, 35, 37].

This is the first study, to our knowledge, investigating the probability of treatment along the patient’s clinical journey while taking the premature death into account and estimating time being alive and treated within the year after the diagnosis. Some previous studies merely compared the mean/median time to treatment across deprivation groups and ignored those who did not receive treatment [28, 34, 35]; some categorised outcomes even if time was involved [29, 35], which may lead to loss of information, and patients did not survive up to treatment would be categorised into no treatment group. Although some studies also used time-to-event analyses [33, 34], it was unclear how the occurrence of death during the follow-up was handled. We use multistate survival models to account for competing risk of death and present both relative and absolute differences in the probability of treatment and death along the follow-up. We also translated our estimates into numbers of days spending in each state to visualise delays in access to treatment and premature death.

Within universal healthcare systems like NHS, every patient expects to receive equal treatment regardless of their socioeconomic status, but we found more deprived patients with colorectal cancer spend less time being alive and treated than the least deprived within the year after diagnosis, even after adjusting for differential clinical and tumour factors. Direct explanations include waiting longer to get treatment and premature death. We speculate that tumour, individual and healthcare factors are contributing to these observed inequalities. First, although we stratified by stage and adjusted for comorbidities and other relevant confounding factors, some stage-independent tumour and individual factors may affect the treatment (e.g. microsatellite instability [38] and performance status [39]), which were not captured in the databases or modelling. Second, as the availability of good medical care (including hospitals with diagnostic and treatment facilities and experienced clinicians) tends to vary inversely with the need for it in the population served—Inverse Care Law remains true within NHS [40, 41], patients from deprived areas are less likely to be in the right care centre in the first place [42], which may cause delays in both diagnosis and treatment after being referred across several hospitals. Third, deprived patients might find it more difficult to navigate within the complex healthcare system and they might not have the same level of social support as their affluent counterparts [43, 44], which will affect patient’s preferences for treatment and ultimately clinicians’ decision-making.

Notably, the events of interest were access to treatment (initiation) and death due to any causes; whether the treatment was completed (in particular, long course radiotherapy or chemotherapy) or the death was the complication of the treatment itself are outside the scope of this study. We have investigated the time to any treatment (surgery, chemotherapy, and radiotherapy) but not the specific modality or quality of care. Future research can provide more insights regarding equitable access to the optimal treatment in patients with colorectal cancer. Use of small-area-based deprivation ranking as a continuous variable or individual income might also provide a better picture of the socioeconomic gradient. In addition, apart from individual factors such as knowledge of cancer, education level etc., accumulating evidence suggests healthcare factors also play a role in these observed inequalities. Therefore, systemic data collection on healthcare system factors could support more research in this area, thereby identifying suitable effective system-level interventions.

In conclusion, our study suggests that, compared to the least deprived quintile, more deprived patients with colon and rectal cancer had a lower probability of receiving treatment and remaining alive, due to both delayed access to treatment and premature death, with larger inequalities in advanced than early stages, and in colon than rectal cancer. These socioeconomic inequalities in treatment may partly explain poorer survival in the more deprived, and should be considered in the cancer policies and other healthcare inequalities improvement programmes. Since COVID-19 pandemic, NHS has reported worst ever waiting time statistics [45], and a recent study showed that reductions in both 2-week-wait referrals and first treatments for cancer were largest in patients from the most deprived areas [46]. These reports suggested that inequalities in access to treatment now are very likely much wider than what we observed in current study. In the general context of the continuing difficulties experienced by the NHS, the issue of care resources available to the most deprived populations deserves to be examined in more detail [47].

## Research in context

### What is already known on this topic

- Summarise the state of scientific knowledge on this subject before you did your study and why this study needed to be done

- Inequalities in colorectal cancer survival were repeatedly reported in England in the past 20 years.
- Individual and tumour factors such as age, stage and comorbidities only partially explain these inequalities.
- Differential management and treatment of colorectal cancer may also contribute to such inequalities.

### What this study adds

- Summarise what we now know as a result of this study that we did not know before

- Compared to the least deprived quintile, the most deprived patients with colon or rectal cancer had a lower probability of being alive and treated (differences ranging from −2.4% to −7.4% in colon cancer and −2.0% to −6.2% in rectal cancer at 6 months after the diagnosis), and a higher probability of being untreated and dead.
- The most deprived spent a smaller number of days being alive and treated (maximum differences observed at 1 year after diagnosis in stage IV colon cancer: 169.3 days in the least deprived vs. 144.7 days in the most deprived) but a greater number of days being untreated and a larger number of days of life lost (earlier death).
- Persistent socioeconomic inequalities in treatment were observed in patients with colorectal cancer, due to both delayed access to treatment and premature death.

### How this study might affect research, practice or policy

- Summarise the implications of this study

Socioeconomic inequalities in treatment may partly explain poorer colon and rectal cancer survival observed in patients from the deprived areas as compared those from the least deprived. Reasons for differential access to treatment should be studied and should be considered in the cancer policy and/or other healthcare inequalities improvement programmes within the National Health Service (NHS). In the general context of the continuing difficulties experienced by the NHS, the issue of care resources available to the most deprived populations deserves to be examined in more detail.

### Supplementary information

Supplemental Material

Supplemental Material STROBE checklist

## Data Availability

Data access is permitted via authorisation from NHS digital only. Clinical code lists and statistical codes are available at GitHub (https://github.com/supingling/colorectal_cancer).
