# Correction: Inequalities in treatment among patients with colon and rectal cancer: a multistate survival model using data from England National Cancer Registry 2012–2016

**DOI:** 10.1038/s41416-023-02486-6

**Published:** 2023-11-14

**Authors:** Suping Ling, Miguel-Angel Luque Fernandez, Manuela Quaresma, Aurelien Belot, Bernard Rachet

**Affiliations:** Inequalities in Cancer Outcome Network (ICON) group, Department of Non-communicable Disease Epidemiology, Faculty of Epidemiology and Population Health, London School of Hygiene & Tropical Medicine, Keppel Street, WC1E 7HT London, UK

**Keywords:** Health care, Cancer, Epidemiology

Correction to: *British Journal of Cancer* 10.1038/s41416-023-02440-6, published online 23 September 2023

In this article table 1 has been given erroneously:

**Table 1:** Baseline characteristics of included patients with colon (*N* = 70,705) or rectal (*N* = 41,991) cancer in England between 2012 and 2016.

*Colon cancer*					
	Total	Stage I	Stage II	Stage III	Stage IV
	*N* = 70,705	*N* = 11,832	*N* = 19,083	*N* = 21,354	*N* = 18,436
Age at diagnosis, years	73.1 (64.4–80.6)	71.2 (62.9–78.7)	74.2 (66.1–81.3)	72.7 (64.2–80.4)	73.2 (63.8–81.4)
Age group, years					
18-44	2727 (3.9%)	734 (6.2%)	625 (3.3%)	707 (3.3%)	661 (3.6%)
45-54	4333 (6.1%)	615 (5.2%)	977 (5.1%)	1398 (6.5%)	1343 (7.3%)
55-64	11,741 (16.6%)	2198 (18.6%)	2709 (14.2%)	3744 (17.5%)	3090 (16.8%)
65-74	21,435 (30.3%)	4076 (34.4%)	5739 (30.1%)	6512 (30.5%)	5108 (27.7%)
74-84	21,875 (30.9%)	3198 (27.0%)	6522 (34.2%)	6549 (30.7%)	5606 (30.4%)
≥85	8594 (12.2%)	1011 (8.5%)	2511 (13.2%)	2444 (11.4%)	2628 (14.3%)
Sex					
Men	37,954 (53.7%)	6662 (56.3%)	10,148 (53.2%)	11,242 (52.6%)	9902 (53.7%)
Women	32,751 (46.3%)	5170 (43.7%)	8935 (46.8%)	10,112 (47.4%)	8534 (46.3%)
Ethnicity					
White	67,221 (95.1%)	11,258 (95.1%)	18,258 (95.7%)	20,219 (94.7%)	17,486 (94.8%)
Other ethnicities	3484 (4.9%)	574 (4.9%)	825 (4.3%)	1135 (5.3%)	950 (5.2%)
Income 2015 quintile					
1 – Least deprived	15,612 (22.1%)	2655 (22.4%)	4211 (22.1%)	4840 (22.7%)	3906 (21.2%)
2	16,374 (23.2%)	2769 (23.4%)	4505 (23.6%)	5005 (23.4%)	4095 (22.2%)
3	14,919 (21.1%)	2454 (20.7%)	4098 (21.5%)	4435 (20.8%)	3932 (21.3%)
4	12,946 (18.3%)	2144 (18.1%)	3498 (18.3%)	3861 (18.1%)	3443 (18.7%)
5 – Most deprived	10,854 (15.4%)	1810 (15.3%)	2771 (14.5%)	3213 (15.0%)	3060 (16.6%)
Comorbidity					
Heart failure	2248 (3.2%)	367 (3.1%)	635 (3.3%)	608 (2.8%)	638 (3.5%)
Myocardial infarction	2966 (4.2%)	488 (4.1%)	891 (4.7%)	857 (4.0%)	730 (4.0%)
Diabetes with complications	529 (0.7%)	94 (0.8%)	141 (0.7%)	139 (0.7%)	155 (0.8%)
Chronic pulmonary disease	8935 (12.6%)	1635 (13.8%)	2488 (13.0%)	2490 (11.7%)	2322 (12.6%)
Route to diagnosis					
Emergency presentation	12,980 (18.4%)	1227 (10.4%)	3225 (16.9%)	3337 (15.6%)	5191 (28.2%)
GP referral	17,798 (25.2%)	3393 (28.7%)	4618 (24.2%)	5370 (25.1%)	4417 (24.0%)
Inpatient elective	2521 (3.6%)	492 (4.2%)	667 (3.5%)	802 (3.8%)	560 (3.0%)
Other outpatient	5221 (7.4%)	959 (8.1%)	1512 (7.9%)	1409 (6.6%)	1,341 (7.3%)
Screening	8754 (12.4%)	2870 (24.3%)	2357 (12.4%)	2762 (12.9%)	765 (4.1%)
TWW	23,431 (33.1%)	2891 (24.4%)	6704 (35.1%)	7674 (35.9%)	6162 (33.4%)
Rectal cancer	Total	Stage I	Stage II	Stage III	Stage IV
	*N* = 41,991	*N* = 9510	*N* = 7504	*N* = 16,346	*N* = 8631
Age at diagnosis, years	70.2 (61.5–78.2)	70.6 (63.0–78.2)	72.6 (63.7–80.1)	68.7 (60.2–77.0)	70.4 (60.6–78.8)
Age group, years					
18–44	1401 (3.3%)	232 (2.4%)	184 (2.5%)	646 (4.0%)	339 (3.9%)
45–54	3723 (8.9%)	675 (7.1%)	521 (6.9%)	1677 (10.3%)	850 (9.8%)
55–64	9076 (21.6%)	1969 (20.7%)	1392 (18.6%)	3890 (23.8%)	1825 (21.1%)
65–74	13,217 (31.5%)	3398 (35.7%)	2237 (29.8%)	5113 (31.3%)	2469 (28.6%)
74–84	11,067 (26.4%)	2460 (25.9%)	2341 (31.2%)	3982 (24.4%)	2284 (26.5%)
≥85	3507 (8.4%)	776 (8.2%)	829 (11.0%)	1038 (6.4%)	864 (10.0%)
Sex					
Male	26,804 (63.8%)	5844 (61.5%)	4815 (64.2%)	10,579 (64.7%)	5566 (64.5%)
Female	15,187 (36.2%)	3666 (38.5%)	2689 (35.8%)	5767 (35.3%)	3065 (35.5%)
Ethnicity					
White	39,935 (95.1%)	9075 (95.4%)	7143 (95.2%)	15,522 (95.0%)	8195 (94.9%)
Other ethnicities	2056 (4.9%)	435 (4.6%)	361 (4.8%)	824 (5.0%)	436 (5.1%)
Income 2015 quintile					
1 – Least deprived	8815 (21.0%)	2138 (22.5%)	1614 (21.5%)	3389 (20.7%)	1674 (19.4%)
2	9488 (22.6%)	2248 (23.6%)	1705 (22.7%)	3705 (22.7%)	1830 (21.2%)
3	8965 (21.3%)	2046 (21.5%)	1564 (20.8%)	3520 (21.5%)	1835 (21.3%)
4	7865 (18.7%)	1685 (17.7%)	1425 (19.0%)	3012 (18.4%)	1743 (20.2%)
5 – Most deprived	6858 (16.3%)	1393 (14.6%)	1196 (15.9%)	2720 (16.6%)	1549 (17.9%)
Comorbidity					
Heart failure	859 (2.0%)	239 (2.5%)	176 (2.3%)	245 (1.5%)	199 (2.3%)
Myocardial infarction	1349 (3.2%)	375 (3.9%)	269 (3.6%)	435 (2.7%)	270 (3.1%)
Diabetes with complications	259 (0.6%)	67 (0.7%)	50 (0.7%)	91 (0.6%)	51 (0.6%)
Chronic pulmonary disease	4292 (10.2%)	1141 (12.0%)	820 (10.9%)	1415 (8.7%)	916 (10.6%)
Route to diagnosis					
Emergency presentation	2920 (7.0%)	356 (3.7%)	522 (7.0%)	791 (4.8%)	1251 (14.5%)
GP referral	11,226 (26.7%)	2969 (31.2%)	1935 (25.8%)	4193 (25.7%)	2129 (24.7%)
Inpatient elective	1557 (3.7%)	400 (4.2%)	261 (3.5%)	599 (3.7%)	297 (3.4%)
Other outpatient	2281 (5.4%)	730 (7.7%)	435 (5.8%)	687 (4.2%)	429 (5.0%)
Screening	5215 (12.4%)	1997 (21.0%)	858 (11.4%)	1927 (11.8%)	433 (5.0%)
TWW	18,792 (44.8%)	3058 (32.2%)	3493 (46.5%)	8149 (49.9%)	4092 (47.4%)

TWW: 2-week-wait referral; comorbidity: patients can have more than one condition listed.

It should be read:

**Table 1:** Baseline characteristics of included patients with colon (*N* = 70,705) or rectal (*N* = 41,991) cancer in England between 2012 and 2016.

*Colon cancer*	Total	Stage I	Stage II	Stage III	Stage IV
	*N* = 70,705	*N* = 11,832	*N* = 19,083	*N* = 21,354	*N* = 18,436
Age at diagnosis, years	73.1 (64.4–80.6)	71.2 (62.9–78.7)	74.2 (66.1–81.3)	72.7 (64.2–80.4)	73.2 (63.8–81.4)
Age group, years					
18-44	2727 (3.9%)	734 (6.2%)	625 (3.3%)	707 (3.3%)	661 (3.6%)
45-54	4333 (6.1%)	615 (5.2%)	977 (5.1%)	1398 (6.5%)	1343 (7.3%)
55-64	11,741 (16.6%)	2198 (18.6%)	2709 (14.2%)	3744 (17.5%)	3090 (16.8%)
65-74	21,435 (30.3%)	4076 (34.4%)	5739 (30.1%)	6512 (30.5%)	5108 (27.7%)
74-84	21,875 (30.9%)	3198 (27.0%)	6522 (34.2%)	6549 (30.7%)	5606 (30.4%)
≥85	8594 (12.2%)	1011 (8.5%)	2511 (13.2%)	2444 (11.4%)	2628 (14.3%)
Sex					
Men	37,954 (53.7%)	6662 (56.3%)	10,148 (53.2%)	11,242 (52.6%)	9902 (53.7%)
Women	32,751 (46.3%)	5170 (43.7%)	8935 (46.8%)	10,112 (47.4%)	8534 (46.3%)
Ethnicity					
White	67,221 (95.1%)	11,258 (95.1%)	18,258 (95.7%)	20,219 (94.7%)	17,486 (94.8%)
Other ethnicities	3484 (4.9%)	574 (4.9%)	825 (4.3%)	1135 (5.3%)	950 (5.2%)
Income 2015 quintile					
1 – Least deprived	15,612 (22.1%)	2655 (22.4%)	4211 (22.1%)	4840 (22.7%)	3906 (21.2%)
2	16,374 (23.2%)	2769 (23.4%)	4505 (23.6%)	5005 (23.4%)	4095 (22.2%)
3	14,919 (21.1%)	2454 (20.7%)	4098 (21.5%)	4435 (20.8%)	3932 (21.3%)
4	12,946 (18.3%)	2144 (18.1%)	3498 (18.3%)	3861 (18.1%)	3443 (18.7%)
5 – Most deprived	10,854 (15.4%)	1810 (15.3%)	2771 (14.5%)	3213 (15.0%)	3060 (16.6%)
Comorbidity					
Heart failure	2248 (3.2%)	367 (3.1%)	635 (3.3%)	608 (2.8%)	638 (3.5%)
Myocardial infarction	2966 (4.2%)	488 (4.1%)	891 (4.7%)	857 (4.0%)	730 (4.0%)
Diabetes with complications	529 (0.7%)	94 (0.8%)	141 (0.7%)	139 (0.7%)	155 (0.8%)
Chronic pulmonary disease	8935 (12.6%)	1635 (13.8%)	2488 (13.0%)	2490 (11.7%)	2322 (12.6%)
Route to diagnosis					
Emergency presentation	12,980 (18.4%)	1227 (10.4%)	3225 (16.9%)	3337 (15.6%)	5191 (28.2%)
GP referral	17,798 (25.2%)	3393 (28.7%)	4618 (24.2%)	5370 (25.1%)	4417 (24.0%)
Inpatient elective	2521 (3.6%)	492 (4.2%)	667 (3.5%)	802 (3.8%)	560 (3.0%)
Other outpatient	5221 (7.4%)	959 (8.1%)	1512 (7.9%)	1409 (6.6%)	1,341 (7.3%)
Screening	8754 (12.4%)	2870 (24.3%)	2357 (12.4%)	2762 (12.9%)	765 (4.1%)
TWW	23,431 (33.1%)	2891 (24.4%)	6704 (35.1%)	7674 (35.9%)	6162 (33.4%)

*Rectal cancer*	Total	Stage I	Stage II	Stage III	Stage IV
	*N* = 41,991	*N* = 9510	*N* = 7504	*N* = 16,346	*N* = 8631
Age at diagnosis, years	70.2 (61.5–78.2)	70.6 (63.0–78.2)	72.6 (63.7–80.1)	68.7 (60.2–77.0)	70.4 (60.6–78.8)
Age group, years					
18–44	1401 (3.3%)	232 (2.4%)	184 (2.5%)	646 (4.0%)	339 (3.9%)
45–54	3723 (8.9%)	675 (7.1%)	521 (6.9%)	1677 (10.3%)	850 (9.8%)
55–64	9076 (21.6%)	1969 (20.7%)	1392 (18.6%)	3890 (23.8%)	1825 (21.1%)
65–74	13,217 (31.5%)	3398 (35.7%)	2237 (29.8%)	5113 (31.3%)	2469 (28.6%)
74–84	11,067 (26.4%)	2460 (25.9%)	2341 (31.2%)	3982 (24.4%)	2284 (26.5%)
≥85	3507 (8.4%)	776 (8.2%)	829 (11.0%)	1038 (6.4%)	864 (10.0%)
Sex					
Male	26,804 (63.8%)	5844 (61.5%)	4815 (64.2%)	10,579 (64.7%)	5566 (64.5%)
Female	15,187 (36.2%)	3666 (38.5%)	2689 (35.8%)	5767 (35.3%)	3065 (35.5%)
Ethnicity					
White	39,935 (95.1%)	9075 (95.4%)	7143 (95.2%)	15,522 (95.0%)	8195 (94.9%)
Other ethnicities	2056 (4.9%)	435 (4.6%)	361 (4.8%)	824 (5.0%)	436 (5.1%)
Income 2015 quintile					
1 – Least deprived	8815 (21.0%)	2138 (22.5%)	1614 (21.5%)	3389 (20.7%)	1674 (19.4%)
2	9488 (22.6%)	2248 (23.6%)	1705 (22.7%)	3705 (22.7%)	1830 (21.2%)
3	8965 (21.3%)	2046 (21.5%)	1564 (20.8%)	3520 (21.5%)	1835 (21.3%)
4	7865 (18.7%)	1685 (17.7%)	1425 (19.0%)	3012 (18.4%)	1743 (20.2%)
5 – Most deprived	6858 (16.3%)	1393 (14.6%)	1196 (15.9%)	2720 (16.6%)	1549 (17.9%)
Comorbidity					
Heart failure	859 (2.0%)	239 (2.5%)	176 (2.3%)	245 (1.5%)	199 (2.3%)
Myocardial infarction	1349 (3.2%)	375 (3.9%)	269 (3.6%)	435 (2.7%)	270 (3.1%)
Diabetes with complications	259 (0.6%)	67 (0.7%)	50 (0.7%)	91 (0.6%)	51 (0.6%)
Chronic pulmonary disease	4292 (10.2%)	1141 (12.0%)	820 (10.9%)	1415 (8.7%)	916 (10.6%)
Route to diagnosis					
Emergency presentation	2920 (7.0%)	356 (3.7%)	522 (7.0%)	791 (4.8%)	1251 (14.5%)
GP referral	11,226 (26.7%)	2969 (31.2%)	1935 (25.8%)	4193 (25.7%)	2129 (24.7%)
Inpatient elective	1557 (3.7%)	400 (4.2%)	261 (3.5%)	599 (3.7%)	297 (3.4%)
Other outpatient	2281 (5.4%)	730 (7.7%)	435 (5.8%)	687 (4.2%)	429 (5.0%)
Screening	5215 (12.4%)	1997 (21.0%)	858 (11.4%)	1927 (11.8%)	433 (5.0%)
TWW	18,792 (44.8%)	3058 (32.2%)	3493 (46.5%)	8149 (49.9%)	4092 (47.4%)

TWW: 2-week-wait referral; comorbidity: patients can have more than one condition listed.

The original article has been corrected.

