# Supplementary material for: Inequalities in treatment among patients with colon and rectal cancer: a multistate survival model using data from England national cancer registry 2012–2016
Source: Br J Cancer. 2023 Sep 23;130(1):88–98. doi: 10.1038/s41416-023-02440-6 (PMC10781675; doi:10.1038/s41416-023-02440-6)
Supplement: Supplementary file 1 — Supplemental Material [file 41416_2023_2440_MOESM1_ESM.pdf]

Supplemental material to

Inequalities in treatment among patients with colon and rectal cancer: a multistate survival model using data from England national cancer registry 2012–2016

Suping Ling, Miguel-Angel Luque, Manuela Quaresma, Aurelien Belot, Bernard Rachet

Contents

Figure S1. Flowchart of patients' selection 2

Figure S2. Numbers of patients entering and staying at each state in patients with colon or rectal cancer by stage (I, II, III, IV, and missing) in England between 2012 and 2016 3

Figure S3. Hazard ratios of socio-economic status and each transition in multistate models in patients with colon or rectal cancer by stage (I, II, III, IV, and missing) in England between 2012 and 2016 4

Figure S4. Probability of being alive and untreated, alive and treated, and dead in patients with missing stage colon or rectal cancer in England between 2012 and 2016 5

Figure S5. Length of stay at alive and untreated, and alive and treated, and days of life lost in patients with missing stage colon (N = 6695) or rectal (N = 2950) cancer in England between 2012 and 2016 6

Figure S6. Probability of staying at alive and untreated, alive and treated, and death in patients with colon cancer by stage in England between 2015 and 2016 7

Figure S7. Probability of staying at alive and untreated, alive and treated, and death in patients with rectal cancer by stage in England between 2015 and 2016 8

Figure S8. Length of stay at alive and untreated, and alive and treated, and days of life lost in patients with colon or rectal cancer by stage in England between 2015 and 2016 9

Figure S9. Probability of staying at alive and untreated, alive and treated, and death in patients with colon cancer by screening stage in England between 2012 and 2016 10

Figure S10. Probability of staying at alive and untreated, alive and treated, and death in patients with rectal cancer by screening stage in England between 2012 and 2016 11

Figure S11. Length of stay at alive and untreated, and alive and treated, and days of life lost in patients with colon or rectal cancer by screening and stage in England between 2012 and 2016 12

Table S1. Missing data patterns in patients with colon or rectal cancer by stage (I, II, III, IV, and missing) in England between 2012 and 2016 13

Table S2. Baseline characteristics of included and excluded patients with colon or rectal cancer in England between 2012 and 2016 14

Table S3. Baseline characteristics of patients with stage missing for colon or rectal cancer in England between 2012 and 2016 15

Table S4. The degree of freedom selection for the baseline hazard function in Royston-Parmar Flexible Parametric model 16

Table S5. Hazard ratios of socioeconomic quintiles for each transition in patients with colorectal cancer by stage in England between 2012 and 2016 17

Table S6. Probability of being alive and untreated, alive and treated or dead in a 75-year-old patient with colon or rectal cancer by stage in England between 2012 and 2016 18

Table S7. Length of staying alive and untreated, and alive and untreated, and days of life lost in patients with colon or rectal cancer by stage in England between 2012 and 2016 20

Figure S1. Flowchart of patients' selection

a) Colon cancer

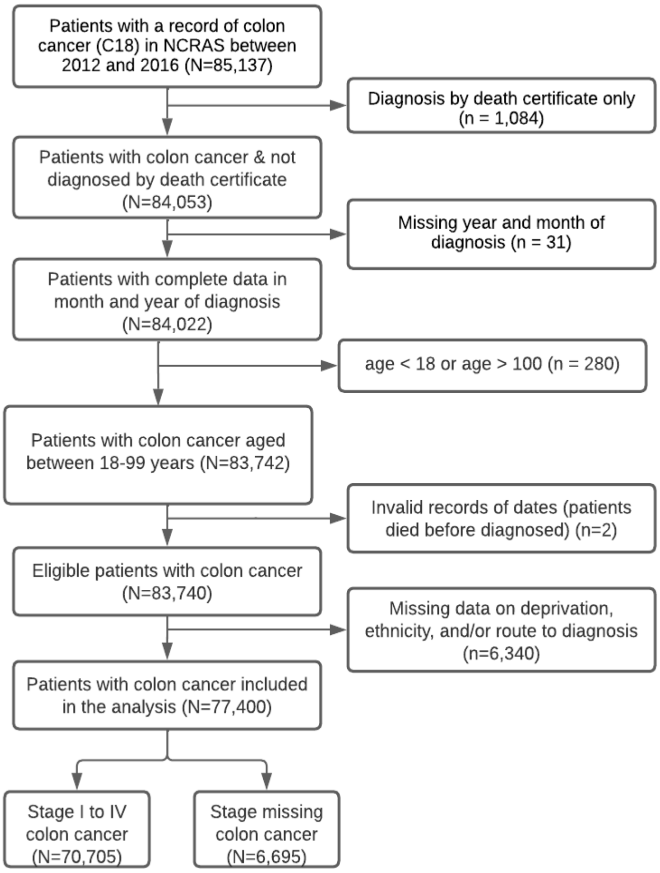

b) Rectal cancer

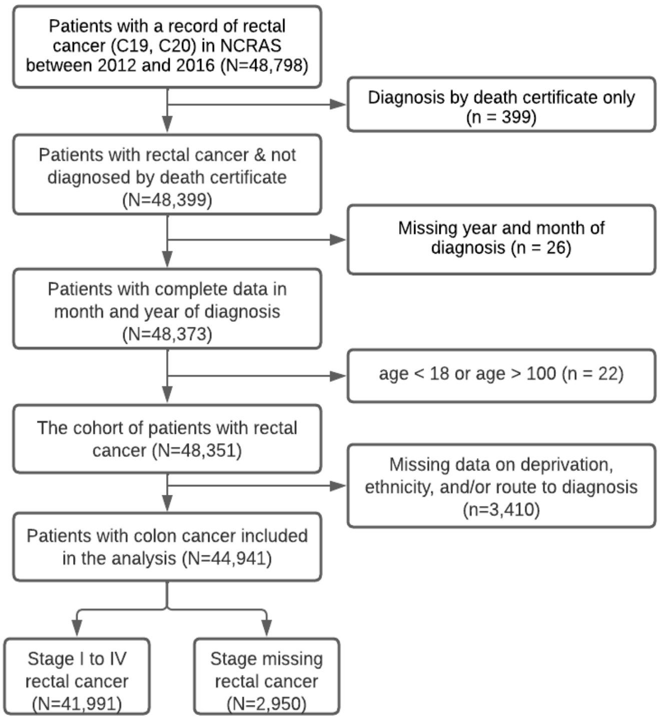

Figure S2. Numbers of patients entering and staying at each state in patients with colon or rectal cancer by stage (I, II, III, IV, and missing) in England between 2012 and 2016

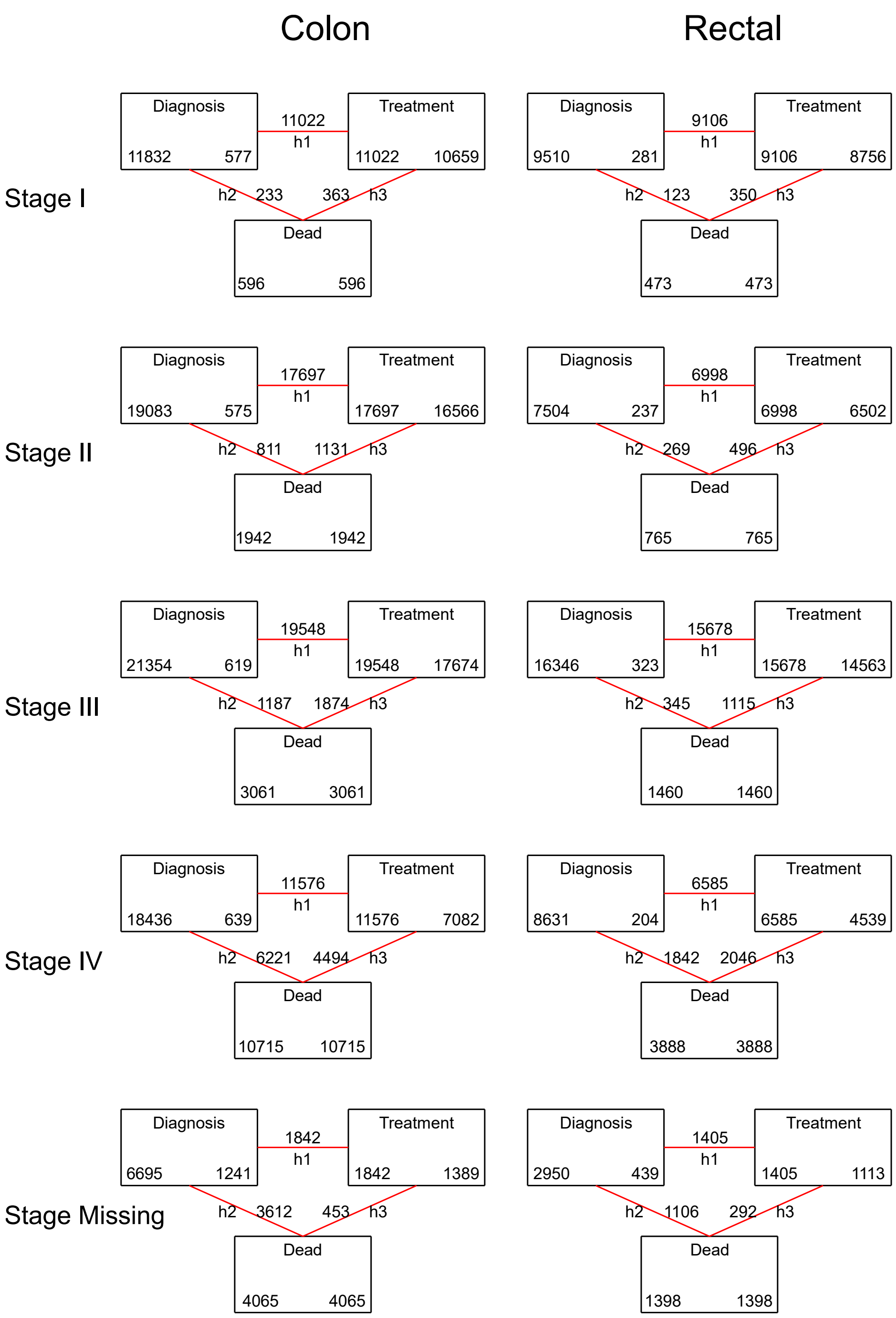

Diagnosis, Treatment, and Dead represent three states where patients can stay during the follow-up. The left number in each box is the number of patients entered this state and the right number is the number of patients stayed at this state at the end of follow-up. Three possible transitions are: h1 diagnosis → treatment, h2 diagnosis → dead, and h3 treatment → dead. Dead is the absorbing state so the number of patients entering and staying at this state is always the same. For example, In stage I colon cancer, 11,832 patients entered "Diagnosis" state (i.e., the total sample), and 577 stayed in this state at the end of the follow-up (i.e. did not receive treatment or die); of 11,255 patients who transited to another state, 11,022 moved to "Treatment" (i.e. received treatment before death) and 233 moved to "Dead" directly (i.e. died before receiving any treatment); of 11,022 patients entered "Treatment" state, 10,659 patients stayed at this state at the end of follow-up and 363 patients transited to "Dead" state (i.e. died after receiving some treatment).

Figure S3. Hazard ratios of socio-economic status and each transition in multistate models in patients with colon or rectal cancer by stage (I, II, III, IV, and missing) in England between 2012 and 2016

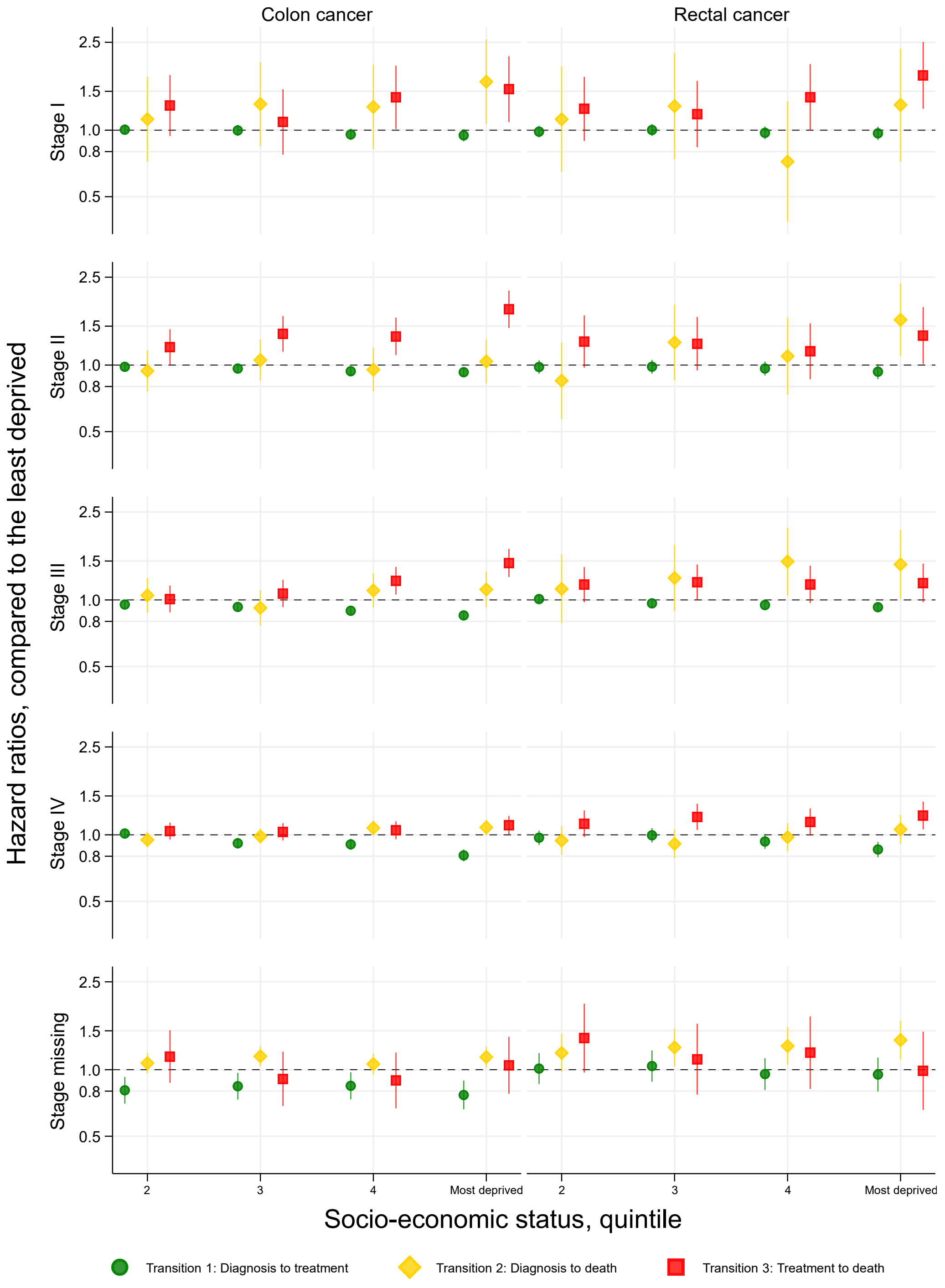

Income 2015 quintile 1 (least deprived) is the reference group for all estimates Transition 1: diagnosis to treatment; transition 2: diagnosis to death; transition 3: treatment to death.

In multistate models, each transition was fitted with a Royston-Parmar flexible parametric survival model and the selection of degree of freedom is shown in Table S3. In all analyses, estimates were adjusted for age (modelled with a cubic spline), sex (men, women), ethnicity (White, ethnicities other than White), heart failure (yes, no), myocardial infarction (yes, no), diabetes with complications (yes, no), chronic pulmonary disease (yes, no), and route to diagnosis (emergency presentation, GP referral, inpatient elective, other outpatient, screening, two-week-wait).

Figure S4. Probability of being alive and untreated, alive and treated, and dead in patients with missing stage colon or rectal cancer in England between 2012 and 2016

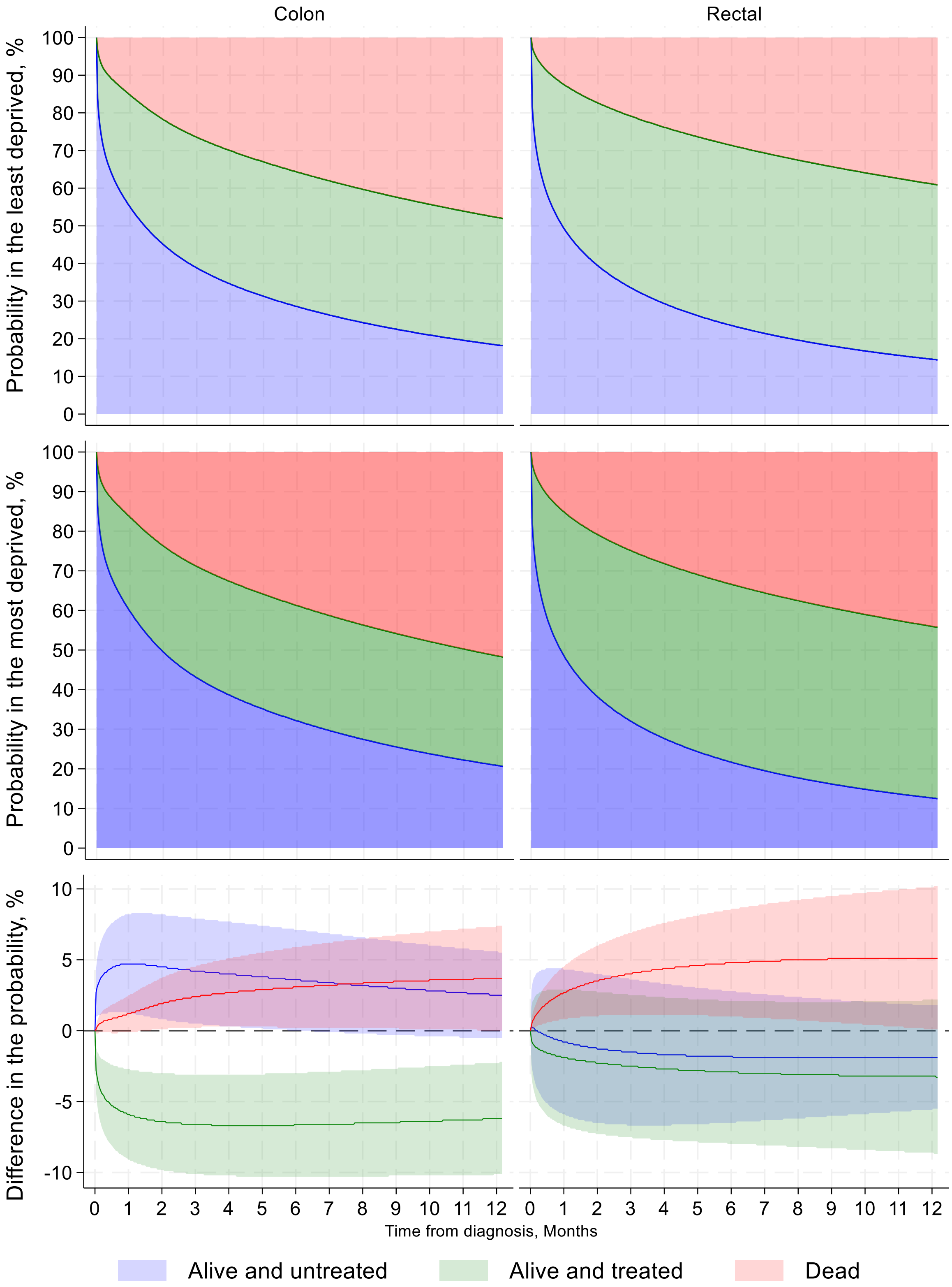

These are fully conditional estimates where socioeconomic status was set at 1st (least deprived) and 5th (most deprived) quintile, age at 75 years old, all other covariates were at their corresponding reference groups (i.e., men, white, no heart failure, no myocardial infarction, no diabetes with complications, no chronic pulmonary disease, and standard GP referral). Difference in the probability was comparing the most deprived to the least deprived.

Figure S5. Length of stay at alive and untreated, and alive and treated, and days of life lost in patients with missing stage colon (N = 6695) or rectal (N = 2950) cancer in England between 2012 and 2016

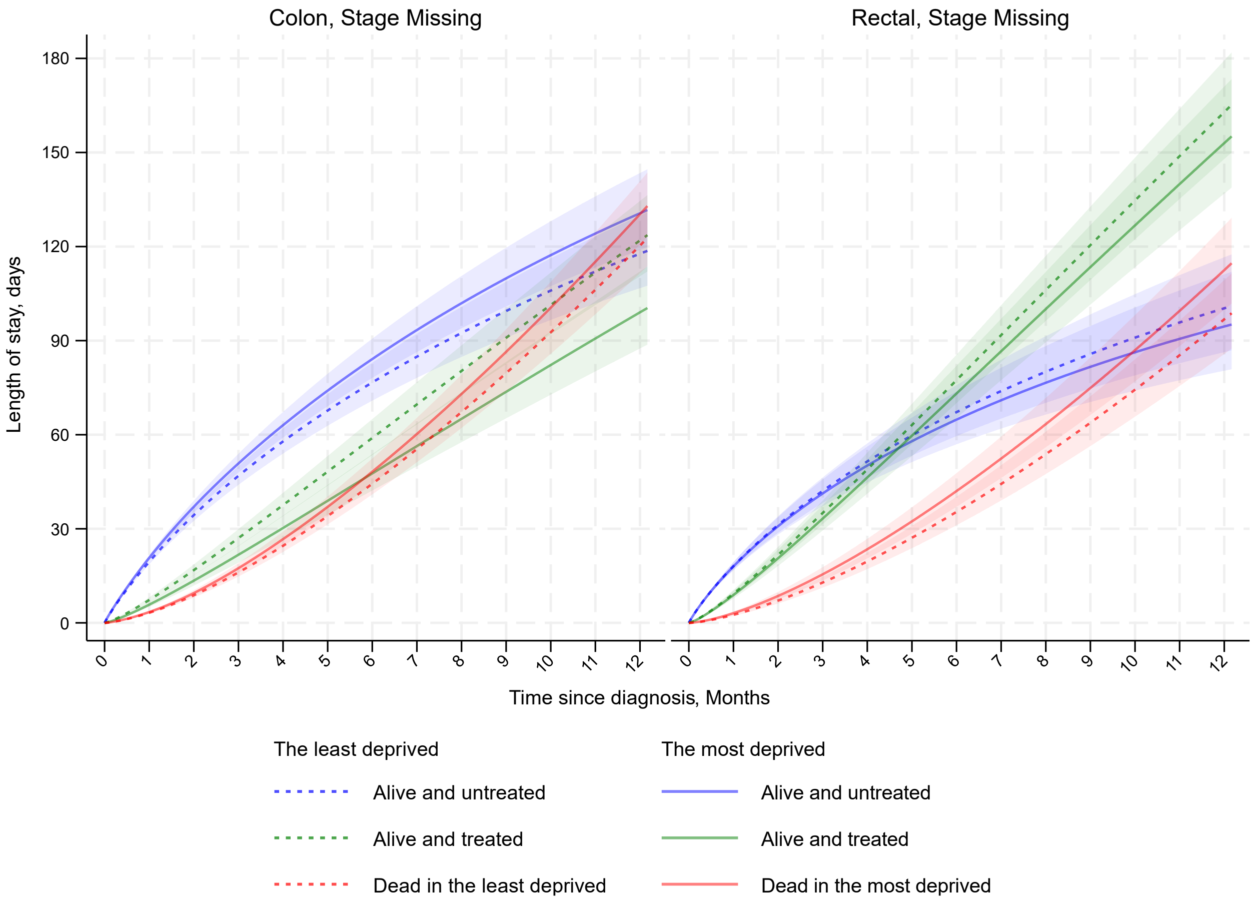

These are fully conditional estimates where socioeconomic status was set at 1st (least deprived) and 5th (most deprived) quintile, age at 75 years old, all other covariates were at their corresponding reference groups (i.e., men, white, no heart failure, no myocardial infarction, no diabetes with complications, no chronic pulmonary disease, and standard referral).

Figure S6. Probability of staying at alive and untreated, alive and treated, and death in patients with colon cancer by stage in England between 2015 and 2016

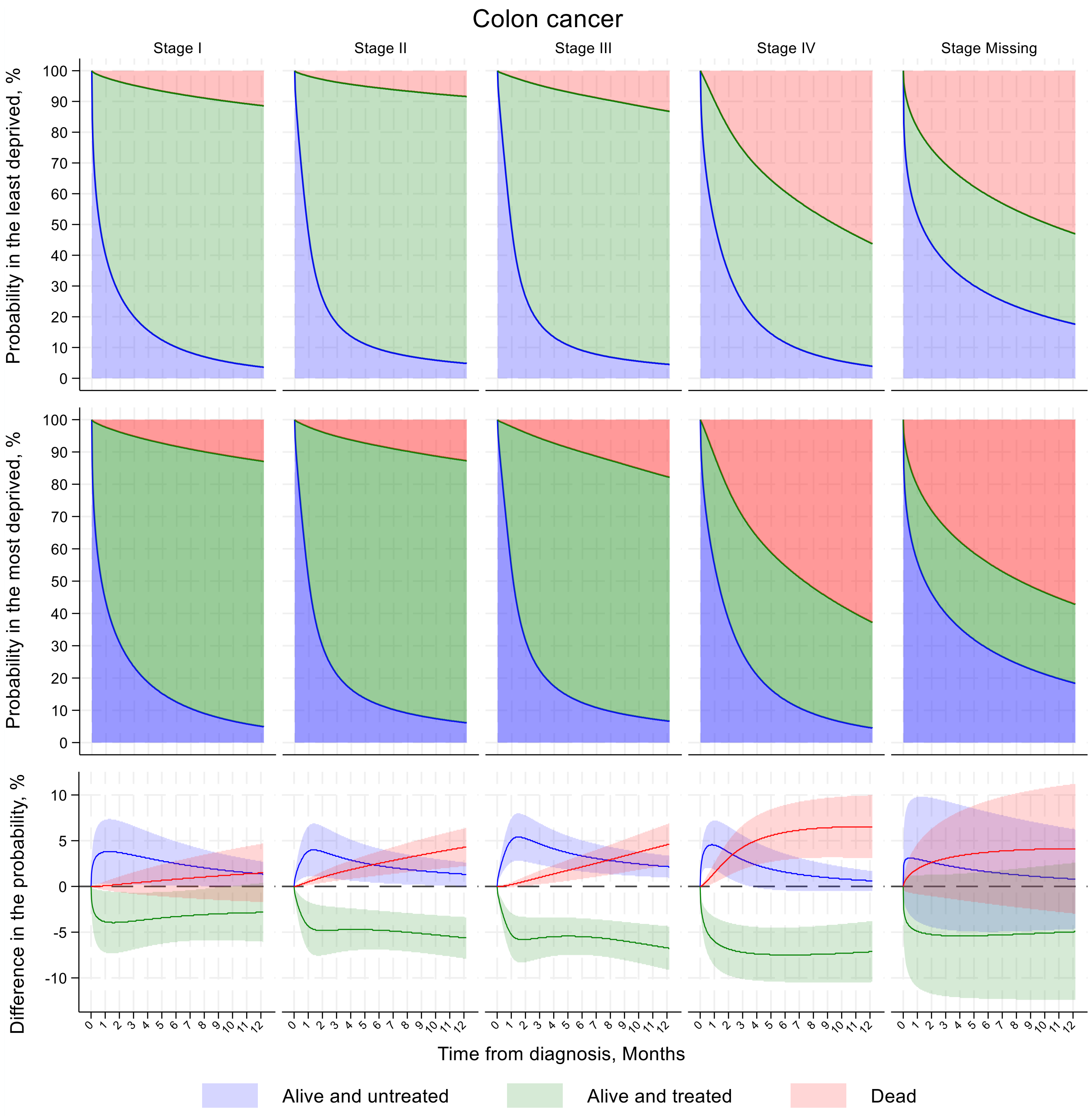

These are fully conditional estimates where socioeconomic status was set at 1st (least deprived) and 5th (most deprived) quintile, age at 75 years old, all other covariates were at their corresponding reference groups (i.e., men, white, no heart failure, no myocardial infarction, no diabetes with complications, no chronic pulmonary disease, and standard referral). Difference in the probability was comparing the most deprived to the least deprived.

Figure S7. Probability of staying at alive and untreated, alive and treated, and death in patients with rectal cancer by stage in England between 2015 and 2016

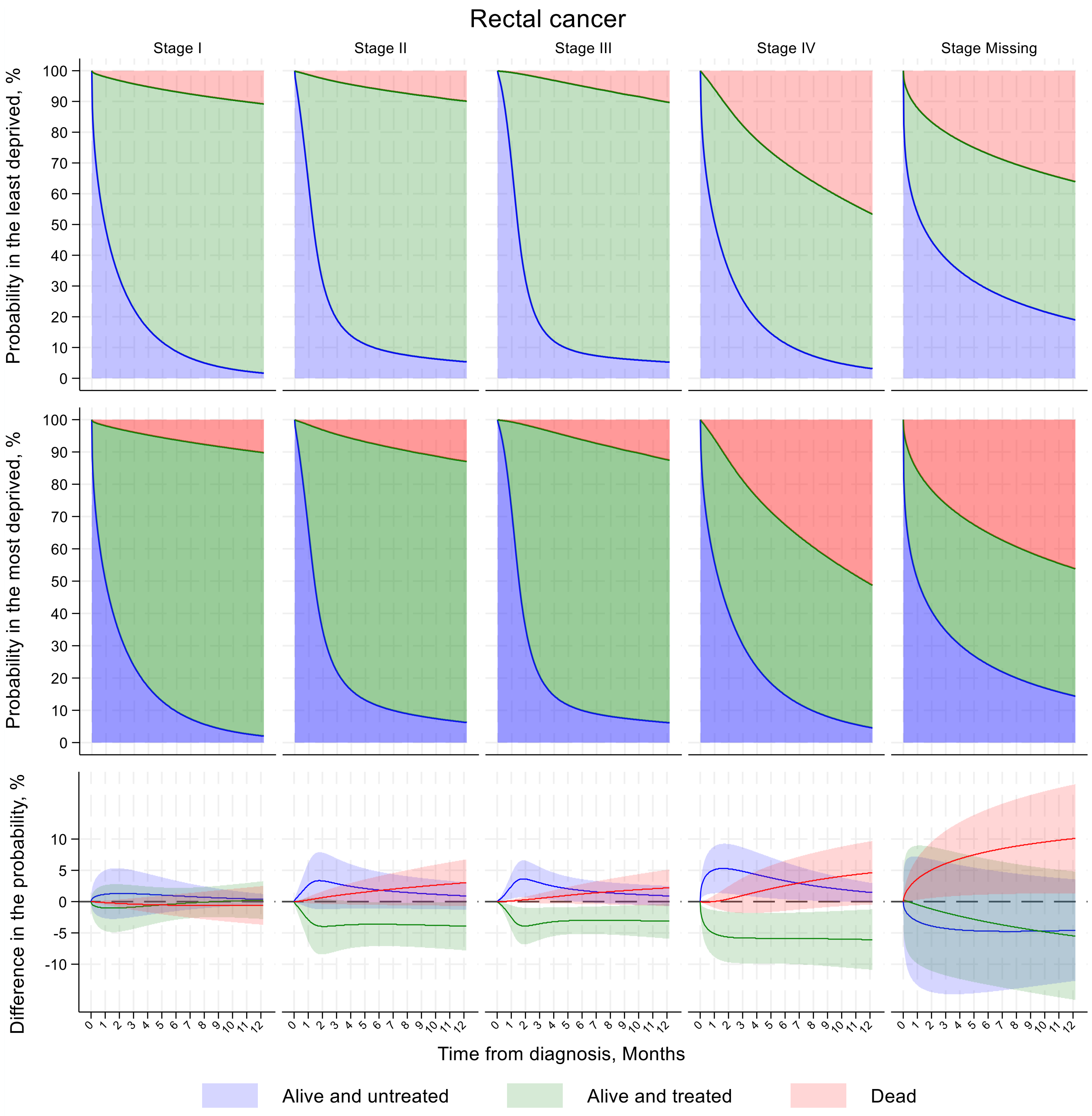

These are fully conditional estimates where socioeconomic status was set at 1st (least deprived) and 5th (most deprived) quintile, age at 75 years old, all other covariates were at their corresponding reference groups (i.e., men, white, no heart failure, no myocardial infarction, no diabetes with complications, no chronic pulmonary disease, and standard referral). Difference in the probability was comparing the most deprived to the least deprived.

Figure S8. Length of stay at alive and untreated, and alive and treated, and days of life lost in patients with colon or rectal cancer by stage in England between 2015 and 2016

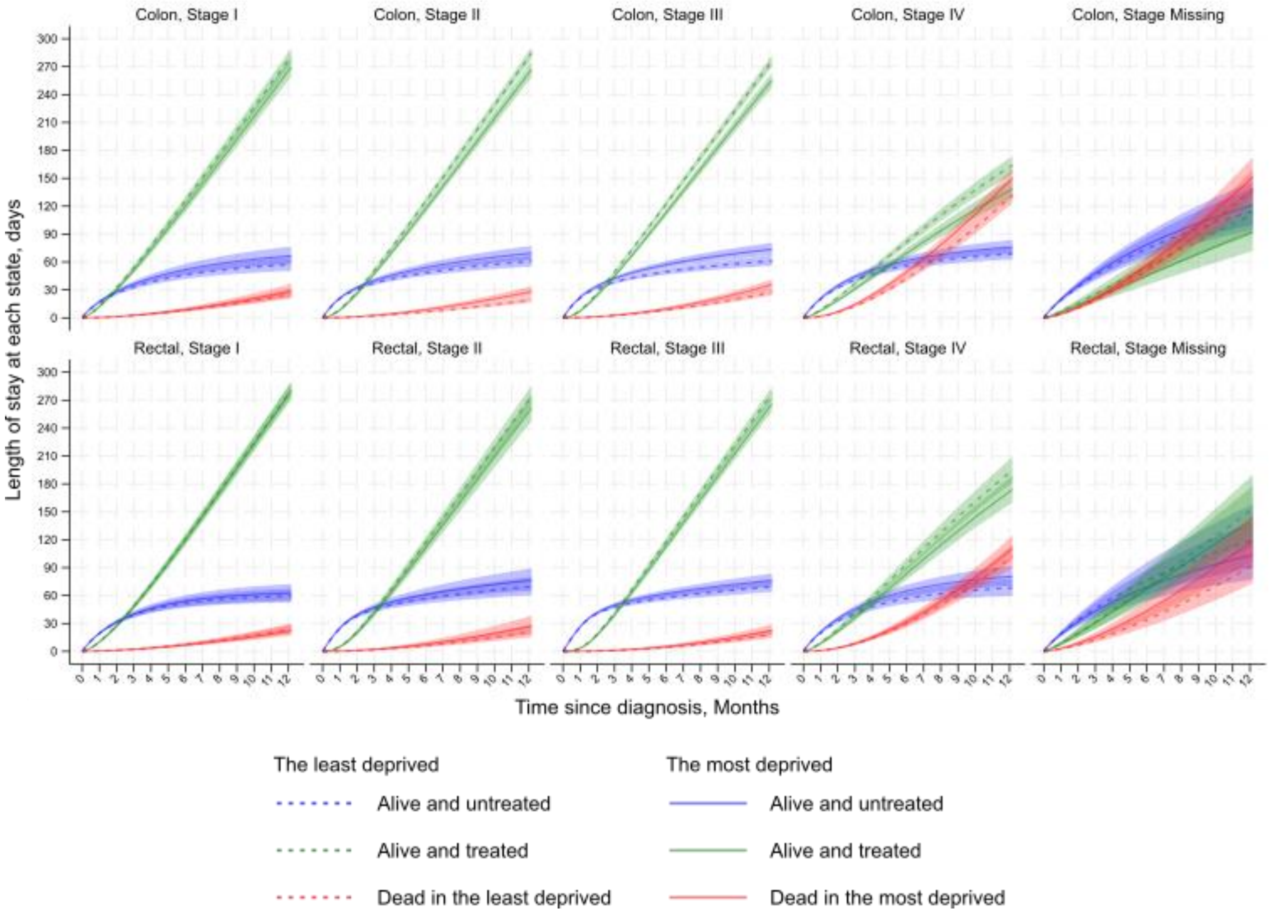

These are fully conditional estimates where socioeconomic status was set at 1st (least deprived) and 5th (most deprived) quintile, age at 75 years old, all other covariates were at their corresponding reference groups (i.e., men, white, no heart failure, no myocardial infarction, no diabetes with complications, no chronic pulmonary disease, and standard referral).

Figure S9. Probability of staying at alive and untreated, alive and treated, and death in patients with colon cancer by screening stage in England between 2012 and 2016

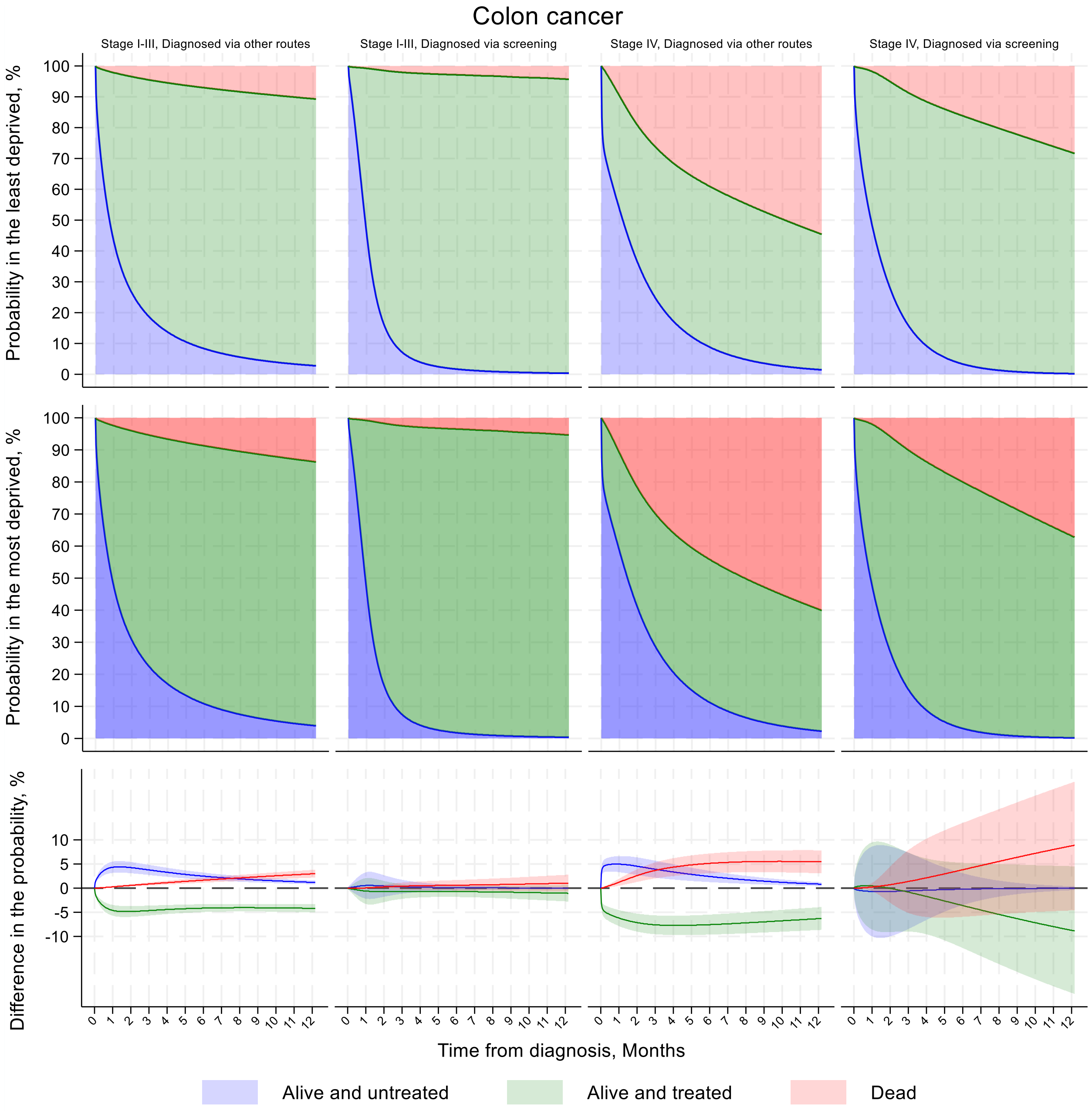

These are fully conditional estimates where socioeconomic status was set at 1st (least deprived) and 5th (most deprived) quintile, age at 75 years old, all other covariates were at their corresponding reference groups (i.e., men, white, no heart failure, no myocardial infarction, no diabetes with complications, no chronic pulmonary disease, and standard referral). Difference in the probability was comparing the most deprived to the least deprived.

Figure S10. Probability of staying at alive and untreated, alive and treated, and death in patients with rectal cancer by screening stage in England between 2012 and 2016

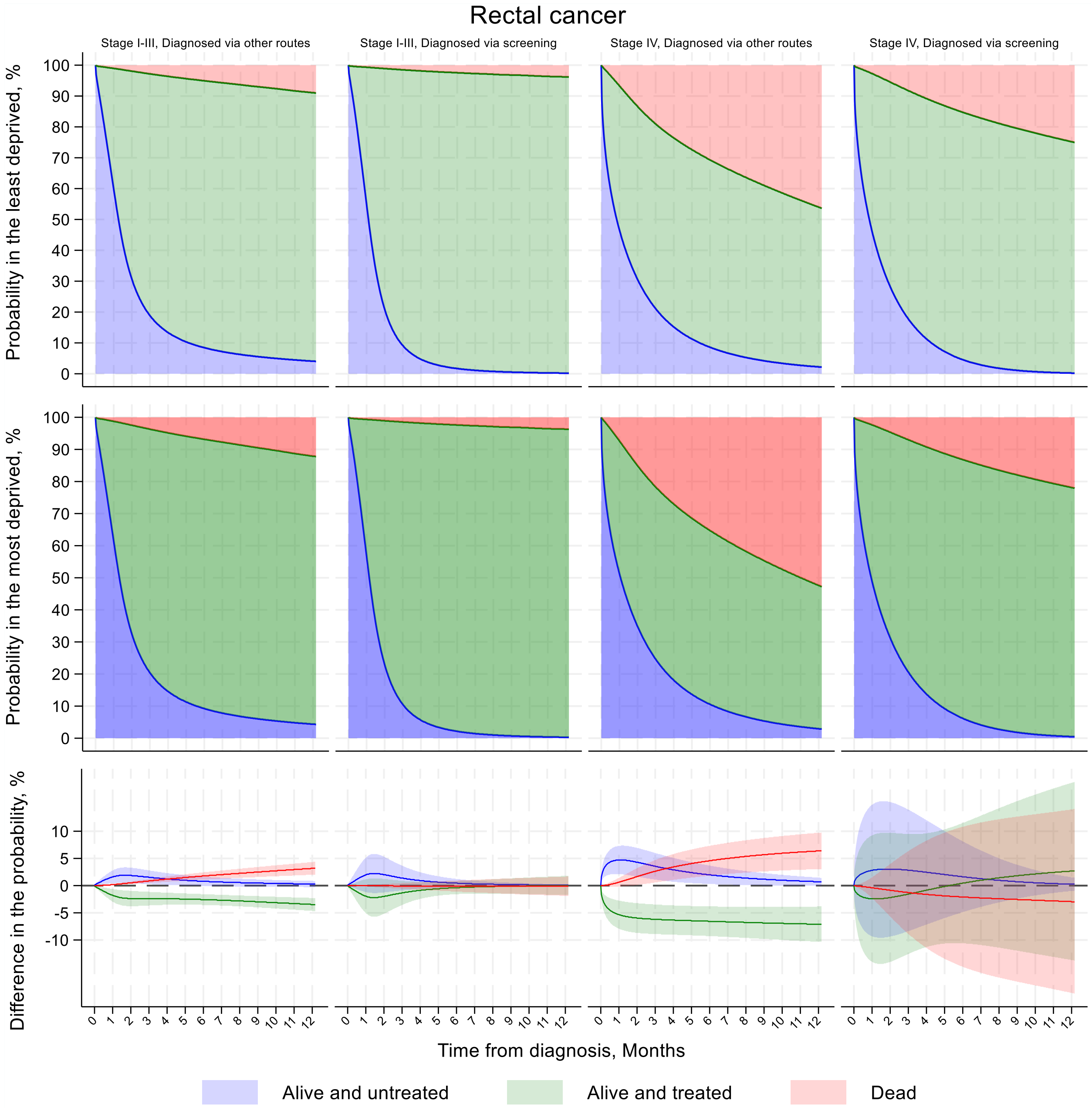

These are fully conditional estimates where socioeconomic status was set at 1st (least deprived) and 5th (most deprived) quintile, age at 75 years old, all other covariates were at their corresponding reference groups (i.e., men, white, no heart failure, no myocardial infarction, no diabetes with complications, no chronic pulmonary disease, and standard referral). Difference in the probability was comparing the most deprived to the least deprived.

Figure S11. Length of stay at alive and untreated, and alive and treated, and days of life lost in patients with colon or rectal cancer by screening and stage in England between 2012 and 2016

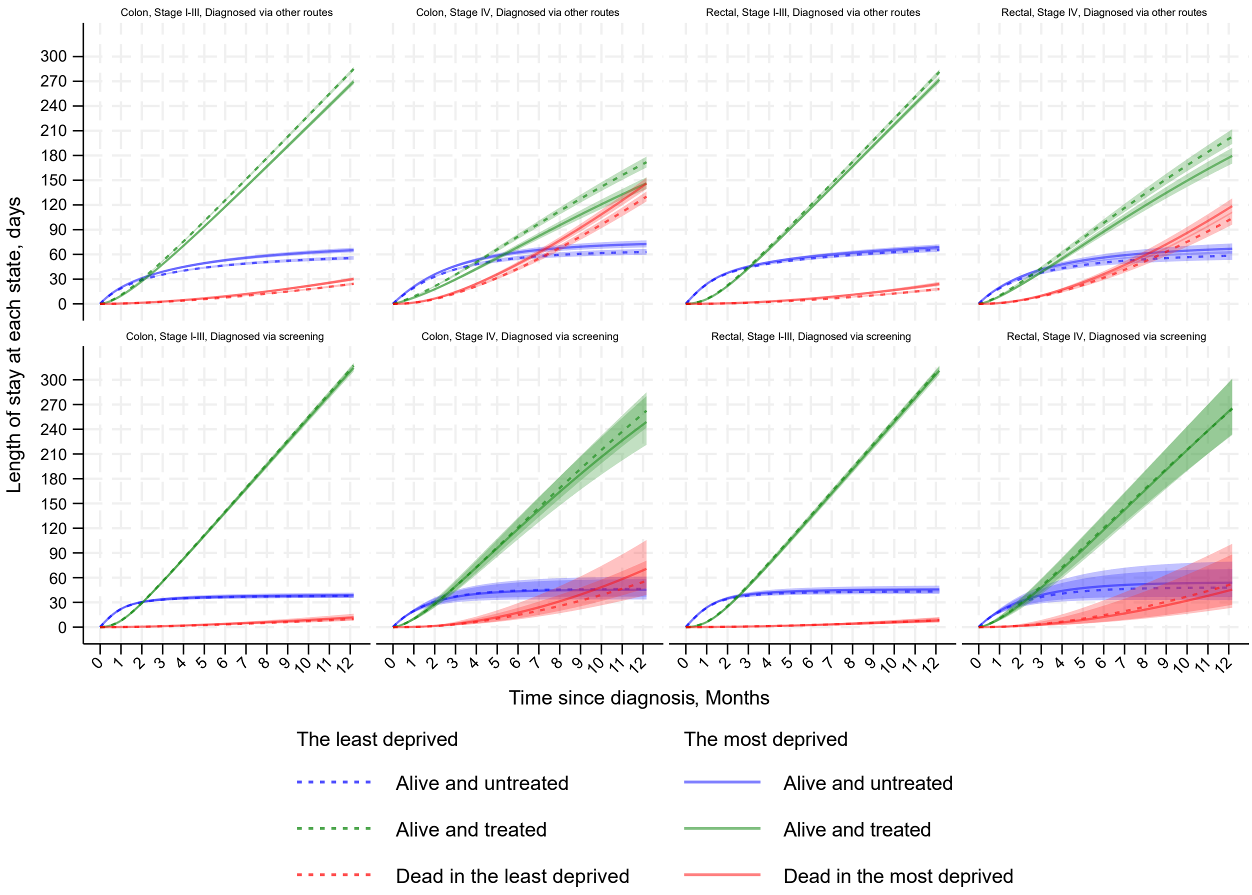

These are fully conditional estimates where socioeconomic status was set at 1st (least deprived) and 5th (most deprived) quintile, age at 75 years old, all other covariates were at their corresponding reference groups (i.e., men, white, no heart failure, no myocardial infarction, no diabetes with complications, no chronic pulmonary disease, and standard referral). Difference in the probability was comparing the most deprived to the least deprived.

Table S1. Missing data patterns in patients with colon or rectal cancer by stage (I, II, III, IV, and missing) in England between 2012 and 2016

Colon cancer	Stage I	Stage II	Stage III	Stage IV	Stage Missing
	N=12,655	N=20,327	N=22,603	N=19,953	N=8,202
Number of covariates missing					
0	11,832 (93.5%)	19,083 (93.9%)	21,354 (94.5%)	18,436 (92.4%)	6,695 (81.6%)
1	754 (6.0%)	1,086 (5.3%)	1,106 (4.9%)	1,363 (6.8%)	1,143 (13.9%)
2	61 (0.5%)	147 (0.7%)	131 (0.6%)	143 (0.7%)	352 (4.3%)
3	8 (0.1%)	11 (0.1%)	12 (0.1%)	11 (0.1%)	12 (0.1%)

Rectal cancer	Stage I	Stage II	Stage III	Stage IV	Stage Missing
	N=10,145	N=7,984	N=17,350	N=9,299	N=3,573
Number of covariates missing					
0	9,510 (93.7%)	7,504 (94.0%)	16,346 (94.2%)	8,631 (92.8%)	2,950 (82.6%)
1	547 (5.4%)	426 (5.3%)	897 (5.2%)	615 (6.6%)	452 (12.7%)
2	79 (0.8%)	46 (0.6%)	96 (0.6%)	48 (0.5%)	165 (4.6%)
3	9 (0.1%)	8 (0.1%)	11 (0.1%)	5 (0.1%)	6 (0.2%)

There was no missing data on age, sex, heart failure, myocardial infarction, diabetes with complications, chronic pulmonary disease or outcomes; potential covariates missing were deprivation (IMD income quintile 2015), ethnicity and route to diagnosis.

Table S2. Baseline characteristics of included and excluded patients with colon or rectal cancer in England between 2012 and 2016

	Colon cancer		Rectal cancer	
	Included<br>N=77,400	Excluded<br>N=6,340	Included<br>N=44,941	Excluded<br>N=3,410
**Stage**				
I	11,832 (15.3%)	823 (13.0%)	9,510 (21.2%)	635 (18.6%)
II	19,083 (24.7%)	1,244 (19.6%)	7,504 (16.7%)	480 (14.1%)
III	21,354 (27.6%)	1,249 (19.7%)	16,346 (36.4%)	1,004 (29.4%)
IV	18,436 (23.8%)	1,517 (23.9%)	8,631 (19.2%)	668 (19.6%)
Missing	6,695 (8.6%)	1,507 (23.8%)	2,950 (6.6%)	623 (18.3%)
**Year of diagnosis**				
2012	15,121 (19.5%)	904 (14.3%)	9,494 (21.1%)	567 (16.6%)
2013	14,787 (19.1%)	975 (15.4%)	8,736 (19.4%)	579 (17.0%)
2014	15,264 (19.7%)	1,189 (18.8%)	8,655 (19.3%)	673 (19.7%)
2015	16,041 (20.7%)	1,623 (25.6%)	8,790 (19.6%)	798 (23.4%)
2016	16,187 (20.9%)	1,649 (26.0%)	9,266 (20.6%)	793 (23.3%)
**Age at diagnosis**				
Median (IQR)	73.8 (64.8-81.5)	72.6 (62.0-82.8)	70.6 (61.9-78.9)	67.6 (58.0-78.3)
18-44	2,864 (3.7%)	380 (6.0%)	1,477 (3.3%)	200 (5.9%)
45-54	4,517 (5.8%)	487 (7.7%)	3,894 (8.7%)	427 (12.5%)
55-64	12,191 (15.8%)	1,109 (17.5%)	9,432 (21.0%)	807 (23.7%)
65-74	22,467 (29.0%)	1,584 (25.0%)	13,742 (30.6%)	917 (26.9%)
74-84	24,039 (31.1%)	1,539 (24.3%)	11,868 (26.4%)	659 (19.3%)
≥85	11,322 (14.6%)	1,241 (19.6%)	4,528 (10.1%)	400 (11.7%)
**Sex**				
Men	41,113 (53.1%)	3,151 (49.7%)	28,407 (63.2%)	2,033 (59.6%)
Women	36,287 (46.9%)	3,189 (50.3%)	16,534 (36.8%)	1,377 (40.4%)
**Ethnicity**				
White	73,681 (95.2%)	1,901 (92.8%)	42,741 (95.1%)	1,196 (94.3%)
Other ethnicities	3,719 (4.8%)	147 (7.2%)	2,200 (4.9%)	72 (5.7%)
Missing	*0*	*4,292 (67.7%)*	*0*	*2,142 (62.8%)*
**Income 2015 quintile**				
1 – Least deprived	16,898 (21.8%)	1,735 (28.0%)	9,360 (20.8%)	1,008 (30.2%)
2	17,801 (23.0%)	1,525 (24.6%)	10,125 (22.5%)	848 (25.4%)
3	16,358 (21.1%)	1,220 (19.7%)	9,592 (21.3%)	648 (19.4%)
4	14,303 (18.5%)	975 (15.7%)	8,480 (18.9%)	480 (14.4%)
5 – Most deprived	12,040 (15.6%)	744 (12.0%)	7,384 (16.4%)	351 (10.5%)
Missing	*0*	*141 (2.2%)*	*0*	*75 (2.2%)*
**Comorbidity**				
Heart failure	3,131 (3.7%)	2,947 (3.8%)	1,067 (2.4%)	29 (0.9%)
Myocardial infarction	3,613 (4.3%)	3,485 (4.5%)	1,512 (3.4%)	36 (1.1%)
Diabetes with complications	647 (0.8%)	625 (0.8%)	295 (0.7%)	6 (0.2%)
Chronic pulmonary disease	10,524 (12.6%)	10,133 (13.1%)	4,735 (10.5%)	137 (4.0%)
**Route to diagnosis**				
Emergency presentation	15,841 (20.5%)	986 (28.2%)	3,769 (8.4%)	205 (12.0%)
GP referral	19,619 (25.3%)	778 (22.3%)	12,166 (27.1%)	406 (23.8%)
Inpatient elective	2,684 (3.5%)	166 (4.8%)	1,655 (3.7%)	111 (6.5%)
Other outpatient	5,788 (7.5%)	208 (6.0%)	2,540 (5.7%)	91 (5.3%)
Screening	9,013 (11.6%)	401 (11.5%)	5,337 (11.9%)	216 (12.7%)
TWW	24,455 (31.6%)	952 (27.3%)	19,474 (43.3%)	676 (39.6%)
Missing	*0*	*2,849 (44.9%)*	*0*	*1,705 (50.0%)*

TWW: two-week-wait referral; comorbidity: patients can have more than one condition listed.

Table S3. Baseline characteristics of patients with stage missing for colon or rectal cancer in England between 2012 and 2016

	Colon cancer	*Rectal cancer*
	N=6,695	N=2,950
**Year of diagnosis**		
2012	1,528 (22.8%)	737 (25.0%)
2013	1,608 (24.0%)	770 (26.1%)
2014	1,387 (20.7%)	602 (20.4%)
2015	1,115 (16.7%)	418 (14.2%)
2016	1,057 (15.8%)	423 (14.3%)
**Age at diagnosis**	82.8 (74.3-88.5)	79.9 (67.9-87.4)
**Age group**		
18-44	137 (2.0%)	76 (2.6%)
45-54	184 (2.7%)	171 (5.8%)
55-64	450 (6.7%)	356 (12.1%)
65-74	1,032 (15.4%)	525 (17.8%)
74-84	2,164 (32.3%)	801 (27.2%)
>=85	2,728 (40.7%)	1,021 (34.6%)
**Sex**		
Men	3,159 (47.2%)	1,603 (54.3%)
Women	3,536 (52.8%)	1,347 (45.7%)
**Ethnicity**		
White	6,460 (96.5%)	2,806 (95.1%)
Other ethnicities	235 (3.5%)	144 (4.9%)
**Income 2015 quintile**		
1 – Least deprived	1,286 (19.2%)	545 (18.5%)
2	1,427 (21.3%)	637 (21.6%)
3	1,439 (21.5%)	627 (21.3%)
4	1,357 (20.3%)	615 (20.8%)
5 – Most deprived	1,186 (17.7%)	526 (17.8%)
**Comorbidity**		
Heart failure	699 (10.4%)	208 (7.1%)
Myocardial infarction	519 (7.8%)	163 (5.5%)
Diabetes with complications	96 (1.4%)	36 (1.2%)
Chronic pulmonary disease	1,198 (17.9%)	443 (15.0%)
**Route to diagnosis**		
Emergency presentation	2,861 (42.7%)	849 (28.8%)
GP referral	1,821 (27.2%)	940 (31.9%)
Inpatient elective	163 (2.4%)	98 (3.3%)
Other outpatient	567 (8.5%)	259 (8.8%)
Screening	259 (3.9%)	122 (4.1%)
TWW	1,024 (15.3%)	682 (23.1%)

TWW: two-week-wait referral; comorbidity: patients can have more than one condition listed.

Table S4. The degree of freedom selection for the baseline hazard function in Royston-Parmar Flexible Parametric model

Cancer	Stage	Transition 1 (h1)				Transition 2 (h2)				Transition 3 (h3)			
		N	AIC	BIC	df	N	AIC	BIC	df	N	AIC	BIC	df
Colon	I	11832	53915	53930	1	11832	1819	1834	1	11022	3942	3957	1
Colon	II	19083	74684	74715	3	19083	5387	5419	3	17697	10999	11030	3
Colon	III	21354	81554	81586	3	21354	6910	6942	3	19548	16000	16032	3
Colon	IV	18436	66646	66678	3	18436	27823	27854	3	11576	23310	23325	1
Colon	Missing	6695	15918	15932	1	6695	20095	20136	5	1842	3035	3046	1
Rectal	I	9510	40288	40309	2	9510	1064	1078	1	9106	3544	3559	1
Rectal	II	7504	25050	25078	3	7504	1947	1961	1	6998	4422	4435	1
Rectal	III	16346	48318	48348	3	16346	2464	2494	3	15678	9562	9584	2
Rectal	IV	8631	35271	35285	1	8631	8905	8933	3	6585	11295	11309	1
Rectal	Missing	2950	10253	10265	1	2950	6616	6628	1	1405	2022	2033	1

N: total sample size; AIC: Akaike Information Criterion; BIC: Bayesian Information Criterion; df: Degree of freedom.

Transition 1 (h1): diagnosis to treatment; transition 2 (h2): diagnosis to death; transition 3 (h3): treatment to death.

Degree of freedom ranging from 1 to 5 were tested where possible, then models with the minimum AIC and/or BIC were selected. Degree of freedom 1 for Royston-Parmar Flexible Parametric model is a Weibull model. In all analyses, models included income quintiles, age (cubic spline transformed), sex, ethnicity, heart failure, myocardial infarction, diabetes with complications, chronic pulmonary disease, and route to diagnosis.

Table S5. Hazard ratios of socioeconomic quintiles for each transition in patients with colorectal cancer by stage in England between 2012 and 2016

Stage	IMD 2015 income quintile	Hazard Ratio (95% confidence interval)					
		Colon cancer			Rectal cancer		
		Transition 1	Transition 2	Transition 3	Transition 1	Transition 2	Transition 3
	Least deprived as reference						
I	2	1.01 (0.95, 1.06)	1.12 (0.72, 1.75)	1.29 (0.94, 1.78)	0.99 (0.93, 1.05)	1.12 (0.65, 1.95)	1.25 (0.89, 1.74)
I	3	1.00 (0.94, 1.06)	1.31 (0.85, 2.04)	1.09 (0.78, 1.53)	1.00 (0.94, 1.07)	1.29 (0.74, 2.24)	1.18 (0.84, 1.67)
I	4	0.96 (0.90, 1.02)	1.27 (0.82, 1.98)	1.41 (1.01, 1.96)	0.97 (0.91, 1.04)	0.72 (0.38, 1.35)	1.41 (1.00, 1.99)
I	Most deprived	0.95 (0.89, 1.01)	1.66 (1.07, 2.58)	1.53 (1.09, 2.16)	0.97 (0.90, 1.04)	1.30 (0.72, 2.35)	1.77 (1.25, 2.51)
	Least deprived as reference						
II	2	0.98 (0.94, 1.03)	0.94 (0.76, 1.16)	1.20 (1.00, 1.45)	0.98 (0.91, 1.05)	0.85 (0.57, 1.27)	1.28 (0.97, 1.68)
II	3	0.96 (0.92, 1.01)	1.05 (0.85, 1.31)	1.38 (1.15, 1.67)	0.98 (0.91, 1.06)	1.27 (0.85, 1.88)	1.25 (0.95, 1.65)
II	4	0.94 (0.90, 0.98)	0.95 (0.76, 1.20)	1.35 (1.11, 1.64)	0.96 (0.90, 1.04)	1.10 (0.73, 1.64)	1.15 (0.86, 1.54)
II	Most deprived	0.93 (0.88, 0.98)	1.04 (0.83, 1.31)	1.79 (1.47, 2.18)	0.93 (0.86, 1.01)	1.60 (1.10, 2.34)	1.36 (1.01, 1.83)
	Least deprived as reference						
III	2	0.95 (0.92, 0.99)	1.05 (0.88, 1.26)	1.01 (0.88, 1.16)	1.01 (0.96, 1.06)	1.12 (0.78, 1.61)	1.17 (0.98, 1.41)
III	3	0.93 (0.89, 0.97)	0.92 (0.77, 1.11)	1.07 (0.93, 1.23)	0.97 (0.92, 1.01)	1.26 (0.89, 1.78)	1.20 (1.00, 1.45)
III	4	0.89 (0.85, 0.93)	1.10 (0.92, 1.32)	1.22 (1.06, 1.41)	0.95 (0.90, 1.00)	1.49 (1.05, 2.13)	1.18 (0.97, 1.43)
III	Most deprived	0.85 (0.81, 0.89)	1.12 (0.93, 1.35)	1.47 (1.27, 1.70)	0.93 (0.88, 0.98)	1.45 (1.01, 2.07)	1.19 (0.98, 1.46)
	Least deprived as reference						
IV	2	1.01 (0.96, 1.07)	0.95 (0.88, 1.03)	1.04 (0.95, 1.13)	0.97 (0.90, 1.05)	0.94 (0.81, 1.09)	1.12 (0.98, 1.29)
IV	3	0.92 (0.87, 0.97)	0.99 (0.91, 1.06)	1.03 (0.94, 1.13)	1.00 (0.92, 1.07)	0.91 (0.78, 1.06)	1.21 (1.05, 1.38)
IV	4	0.91 (0.85, 0.96)	1.07 (0.99, 1.16)	1.05 (0.95, 1.15)	0.93 (0.86, 1.01)	0.98 (0.84, 1.13)	1.14 (0.99, 1.32)
IV	Most deprived	0.81 (0.76, 0.86)	1.08 (1.00, 1.17)	1.10 (1.00, 1.22)	0.86 (0.79, 0.93)	1.06 (0.91, 1.23)	1.22 (1.06, 1.41)
	Least deprived as reference						
Missing	2	0.81 (0.70, 0.93)	1.07 (0.96, 1.19)	1.15 (0.87, 1.51)	1.01 (0.86, 1.19)	1.19 (0.98, 1.45)	1.39 (0.97, 1.99)
Missing	3	0.84 (0.73, 0.97)	1.15 (1.04, 1.28)	0.91 (0.69, 1.21)	1.04 (0.88, 1.22)	1.26 (1.03, 1.54)	1.12 (0.77, 1.61)
Missing	4	0.85 (0.73, 0.97)	1.06 (0.95, 1.18)	0.89 (0.67, 1.20)	0.96 (0.81, 1.13)	1.28 (1.05, 1.56)	1.19 (0.82, 1.74)
Missing	Most deprived	0.77 (0.66, 0.89)	1.14 (1.02, 1.28)	1.05 (0.78, 1.41)	0.95 (0.80, 1.14)	1.36 (1.11, 1.67)	0.99 (0.66, 1.49)

Transition 1: diagnosis to treatment; transition 2: diagnosis to death; transition 3: treatment to death. In all analyses, estimates were adjusted for age (modelled with a cubic spline), sex (men, women), ethnicity (White, ethnicities other than White), heart failure (yes, no), myocardial infarction (yes, no), diabetes with complications (yes, no), chronic pulmonary disease (yes, no), and route to diagnosis (emergency presentation, GP referral, inpatient elective, other outpatient, screening, two-week-wait). These estimates are also shown in Figure S3.

Table S6. Probability of being alive and untreated, alive and treated or dead in a 75-year-old patient with colon or rectal cancer by stage in England between 2012 and 2016

Follow-up, days	Probability of being alive and untreated, %			Probability of being alive and treated, %			Probability of death, %		
	Least deprived	Most deprived	Difference	Least deprived	Most deprived	Difference	Least deprived	Most deprived	Difference
**Colon**									
**Stage I**									
1 month	40.3 (38.3, 42.4)	42.2 (39.9, 44.5)	1.8 (-0.4, 4.1)	57.8 (55.8, 59.7)	55.7 (53.5, 57.9)	-2.1 (-4.3, 0.1)	1.9 (1.7, 2.1)	2.2 (1.9, 2.5)	0.2 (0.0, 0.5)
2 months	27.7 (25.8, 29.7)	29.4 (27.2, 31.7)	1.7 (-0.5, 4.0)	69.3 (67.4, 71.1)	67.1 (64.9, 69.2)	-2.2 (-4.4, -0.1)	3.0 (2.7, 3.3)	3.5 (3.0, 4.0)	0.5 (0.1, 0.9)
3 months	20.7 (19.0, 22.6)	22.2 (20.2, 24.4)	1.5 (-0.5, 3.6)	75.4 (73.7, 77.1)	73.1 (71.1, 75.1)	-2.3 (-4.2, -0.3)	3.9 (3.4, 4.4)	4.6 (4.0, 5.3)	0.8 (0.2, 1.3)
6 months	10.7 (9.4, 12.1)	11.7 (10.2, 13.3)	1.0 (-0.6, 2.5)	83.3 (81.9, 84.6)	80.9 (79.2, 82.6)	-2.4 (-4.0, -0.8)	6.0 (5.3, 6.8)	7.4 (6.4, 8.6)	1.4 (0.4, 2.4)
12 months	4.1 (3.4, 4.9)	4.5 (3.7, 5.5)	0.4 (-0.5, 1.3)	86.9 (85.6, 88.1)	84.2 (82.4, 85.8)	-2.7 (-4.3, -1.1)	9.0 (8.0, 10.2)	11.3 (9.7, 13.2)	2.3 (0.7, 3.9)
**Stage II**									
1 month	45.0 (43.4, 46.6)	47.6 (45.7, 49.4)	2.6 (0.8, 4.4)	53.0 (51.5, 54.6)	50.0 (48.2, 51.8)	-3.0 (-4.7, -1.3)	2.0 (1.8, 2.2)	2.4 (2.1, 2.7)	0.4 (0.2, 0.7)
2 months	23.9 (22.4, 25.5)	26.4 (24.7, 28.3)	2.5 (0.8, 4.2)	72.9 (71.4, 74.4)	69.6 (67.8, 71.3)	-3.3 (-5.0, -1.6)	3.2 (2.8, 3.5)	4.0 (3.5, 4.5)	0.8 (0.4, 1.2)
3 months	15.9 (14.6, 17.2)	18.0 (16.5, 19.7)	2.1 (0.6, 3.7)	80.0 (78.7, 81.3)	76.8 (75.1, 78.3)	-3.3 (-4.8, -1.8)	4.1 (3.7, 4.6)	5.2 (4.6, 5.9)	1.1 (0.6, 1.7)
6 months	7.7 (6.9, 8.7)	9.2 (8.1, 10.4)	1.4 (0.4, 2.5)	86.2 (85.1, 87.2)	82.9 (81.4, 84.2)	-3.3 (-4.5, -2.0)	6.1 (5.5, 6.8)	7.9 (7.0, 9.0)	1.8 (1.0, 2.7)
12 months	3.6 (3.0, 4.3)	4.5 (3.7, 5.3)	0.9 (0.2, 1.5)	87.9 (86.8, 88.9)	84.1 (82.5, 85.5)	-3.8 (-5.1, -2.5)	8.5 (7.6, 9.5)	11.5 (10.1, 13.0)	2.9 (1.7, 4.2)
**Stage III**									
1 month	47.1 (45.5, 48.6)	52.5 (50.8, 54.2)	5.4 (3.8, 7.0)	51.0 (49.5, 52.4)	45.3 (43.7, 47.0)	-5.6 (-7.2, -4.1)	2.0 (1.8, 2.2)	2.2 (1.9, 2.4)	0.2 (0.0, 0.4)
2 months	24.5 (23.1, 26.0)	30.0 (28.3, 31.8)	5.5 (3.8, 7.1)	72.0 (70.6, 73.5)	66.0 (64.3, 67.7)	-6.0 (-7.6, -4.4)	3.4 (3.1, 3.8)	4.0 (3.6, 4.4)	0.6 (0.2, 0.9)
3 months	16.1 (14.9, 17.4)	20.9 (19.3, 22.5)	4.7 (3.3, 6.2)	79.2 (77.9, 80.4)	73.5 (71.9, 75.1)	-5.7 (-7.1, -4.2)	4.7 (4.3, 5.1)	5.6 (5.1, 6.2)	0.9 (0.4, 1.4)
6 months	7.9 (7.0, 8.8)	11.0 (9.8, 12.2)	3.1 (2.1, 4.2)	84.5 (83.4, 85.5)	79.3 (77.8, 80.7)	-5.2 (-6.4, -3.9)	7.7 (7.0, 8.4)	9.7 (8.8, 10.8)	2.0 (1.2, 2.9)
12 months	3.7 (3.2, 4.4)	5.5 (4.7, 6.5)	1.8 (1.1, 2.5)	83.6 (82.3, 84.8)	77.8 (76.0, 79.5)	-5.8 (-7.3, -4.3)	12.7 (11.5, 13.9)	16.6 (15.0, 18.4)	4.0 (2.5, 5.4)
**Stage IV**									
1 month	54.4 (52.8, 56.0)	59.0 (57.4, 60.7)	4.7 (3.0, 6.3)	36.0 (34.4, 37.6)	30.2 (28.6, 31.8)	-5.8 (-7.4, -4.2)	9.6 (8.9, 10.3)	10.8 (10.0, 11.7)	1.2 (0.5, 1.9)
2 months	36.8 (35.1, 38.5)	41.0 (39.2, 42.9)	4.3 (2.5, 6.1)	44.0 (42.2, 45.8)	37.2 (35.3, 39.0)	-6.8 (-8.6, -5.0)	19.3 (18.1, 20.5)	21.8 (20.4, 23.3)	2.6 (1.2, 3.9)
3 months	24.8 (23.2, 26.4)	28.5 (26.7, 30.3)	3.7 (1.9, 5.4)	48.8 (47.0, 50.6)	41.5 (39.6, 43.4)	-7.3 (-9.2, -5.4)	26.4 (24.9, 28.0)	30.0 (28.3, 31.9)	3.6 (2.0, 5.3)
6 months	8.6 (7.7, 9.7)	10.9 (9.7, 12.2)	2.3 (1.1, 3.4)	52.2 (50.3, 54.1)	44.8 (42.7, 46.9)	-7.4 (-9.4, -5.3)	39.2 (37.3, 41.1)	44.3 (42.1, 46.4)	5.1 (3.1, 7.1)
12 months	1.4 (1.2, 1.8)	2.2 (1.8, 2.7)	0.8 (0.4, 1.1)	44.3 (42.1, 46.6)	38.0 (35.7, 40.4)	-6.3 (-8.6, -4.0)	54.2 (52.0, 56.4)	59.8 (57.3, 62.1)	5.5 (3.2, 7.9)
**Stage missing**									
1 month	55.1 (51.7, 58.4)	59.8 (56.4, 63.1)	4.7 (1.2, 8.2)	29.7 (26.7, 32.8)	23.8 (20.9, 26.9)	-5.9 (-9.1, -2.7)	15.2 (13.9, 16.6)	16.4 (14.9, 18.0)	1.2 (-0.1, 2.5)
2 months	45.0 (41.5, 48.6)	49.5 (45.9, 53.1)	4.5 (0.8, 8.2)	33.2 (30.0, 36.5)	26.8 (23.6, 30.2)	-6.4 (-9.8, -3.0)	21.8 (20.0, 23.7)	23.7 (21.7, 25.9)	1.9 (0.1, 3.7)
3 months	38.8 (35.4, 42.4)	43.0 (39.4, 46.8)	4.2 (0.5, 7.9)	34.7 (31.4, 38.1)	28.1 (24.8, 31.6)	-6.6 (-10.1, -3.1)	26.5 (24.4, 28.7)	28.9 (26.5, 31.4)	2.4 (0.2, 4.5)
6 months	28.6 (25.3, 32.0)	32.1 (28.7, 35.8)	3.6 (0.0, 7.1)	35.7 (32.3, 39.3)	29.1 (25.7, 32.8)	-6.6 (-10.3, -3.0)	35.7 (33.0, 38.5)	38.8 (35.7, 41.9)	3.1 (0.2, 5.9)
12 months	18.3 (15.7, 21.3)	20.9 (17.9, 24.2)	2.5 (-0.5, 5.6)	33.9 (30.2, 37.8)	27.7 (24.0, 31.7)	-6.2 (-10.1, -2.3)	47.8 (44.2, 51.3)	51.5 (47.7, 55.2)	3.7 (0.0, 7.4)

Follow-up, days	Probability of being alive and untreated, %			Probability of being alive and treated, %			Probability of death, %		
	Least deprived	Most deprived	Difference	Least deprived	Most deprived	Difference	Least deprived	Most deprived	Difference
**Rectal**									
**Stage I**									
1 month	49.6 (47.5, 51.8)	50.7 (48.2, 53.1)	1.1 (-1.3, 3.5)	48.6 (46.6, 50.7)	47.4 (45.1, 49.8)	-1.2 (-3.5, 1.1)	1.7 (1.5, 2.0)	1.9 (1.6, 2.2)	0.1 (0.0, 0.3)
2 months	32.7 (30.5, 34.9)	33.8 (31.3, 36.4)	1.1 (-1.4, 3.6)	64.7 (62.5, 66.7)	63.2 (60.7, 65.6)	-1.5 (-3.9, 0.9)	2.7 (2.3, 3.1)	3.1 (2.6, 3.6)	0.4 (0.0, 0.8)
3 months	22.4 (20.5, 24.5)	23.4 (21.2, 25.9)	1.0 (-1.4, 3.3)	74.1 (72.1, 75.9)	72.4 (70.1, 74.6)	-1.6 (-3.8, 0.6)	3.5 (3.0, 4.0)	4.1 (3.5, 4.9)	0.6 (0.1, 1.2)
6 months	8.2 (7.1, 9.5)	8.8 (7.4, 10.4)	0.6 (-0.9, 2.0)	86.4 (85.0, 87.6)	84.4 (82.7, 86.0)	-2.0 (-3.5, -0.4)	5.4 (4.7, 6.3)	6.8 (5.8, 8.1)	1.4 (0.3, 2.4)
12 months	1.4 (1.1, 1.9)	1.6 (1.2, 2.1)	0.1 (-0.3, 0.6)	90.3 (89.0, 91.5)	87.5 (85.5, 89.3)	-2.8 (-4.6, -1.0)	8.3 (7.1, 9.6)	10.9 (9.1, 13.0)	2.7 (0.9, 4.5)
**Stage II**									
1 month	63.3 (61.2, 65.4)	65.0 (62.7, 67.3)	1.7 (-0.5, 3.9)	35.6 (33.5, 37.7)	33.5 (31.3, 35.8)	-2.0 (-4.2, 0.1)	1.1 (0.9, 1.4)	1.4 (1.1, 1.8)	0.3 (0.1, 0.6)
2 months	31.0 (28.5, 33.6)	33.1 (30.3, 36.1)	2.1 (-0.7, 5.0)	66.9 (64.3, 69.4)	63.9 (61.0, 66.8)	-2.9 (-5.7, -0.1)	2.2 (1.7, 2.7)	2.9 (2.3, 3.7)	0.8 (0.2, 1.4)
3 months	18.6 (16.5, 20.9)	20.4 (17.9, 23.0)	1.8 (-0.7, 4.3)	78.3 (76.0, 80.5)	75.3 (72.6, 77.9)	-3.0 (-5.5, -0.5)	3.1 (2.5, 3.8)	4.3 (3.4, 5.3)	1.2 (0.4, 2.0)
6 months	9.0 (7.6, 10.7)	10.1 (8.4, 12.1)	1.0 (-0.7, 2.8)	85.5 (83.6, 87.2)	82.3 (79.9, 84.5)	-3.2 (-5.3, -1.1)	5.5 (4.5, 6.6)	7.6 (6.2, 9.3)	2.1 (0.7, 3.5)
12 months	5.4 (4.3, 6.9)	5.8 (4.4, 7.5)	0.3 (-1.0, 1.6)	85.5 (83.3, 87.4)	81.6 (78.7, 84.1)	-3.9 (-6.3, -1.5)	9.1 (7.5, 11.1)	12.7 (10.3, 15.4)	3.6 (1.3, 5.9)
**Stage III**									
1 month	68.5 (67.2, 69.9)	70.3 (68.9, 71.7)	1.8 (0.5, 3.1)	30.8 (29.6, 32.2)	28.9 (27.6, 30.3)	-1.9 (-3.2, -0.6)	0.6 (0.5, 0.7)	0.7 (0.6, 0.9)	0.1 (0.0, 0.2)
2 months	30.4 (28.7, 32.2)	32.9 (31.0, 34.9)	2.5 (0.6, 4.4)	68.1 (66.3, 69.8)	65.2 (63.3, 67.1)	-2.9 (-4.8, -1.0)	1.5 (1.2, 1.8)	1.9 (1.5, 2.3)	0.4 (0.1, 0.7)
3 months	16.0 (14.7, 17.5)	18.0 (16.5, 19.7)	2.0 (0.4, 3.5)	81.5 (80.0, 82.9)	78.8 (77.1, 80.4)	-2.7 (-4.3, -1.1)	2.5 (2.1, 2.9)	3.2 (2.6, 3.8)	0.7 (0.2, 1.2)
6 months	6.7 (5.8, 7.6)	7.6 (6.6, 8.8)	1.0 (0.0, 2.0)	88.0 (86.8, 89.1)	85.5 (84.1, 86.9)	-2.5 (-3.8, -1.2)	5.3 (4.5, 6.2)	6.8 (5.8, 8.1)	1.5 (0.5, 2.6)
12 months	4.2 (3.4, 5.0)	4.6 (3.8, 5.6)	0.4 (-0.4, 1.3)	85.5 (83.9, 87.1)	82.5 (80.5, 84.3)	-3.1 (-4.9, -1.3)	10.3 (8.9, 11.9)	12.9 (11.1, 14.9)	2.6 (0.8, 4.5)
**Stage IV**									
1 month	47.1 (44.7, 49.6)	51.6 (49.0, 54.1)	4.4 (1.9, 6.9)	46.2 (43.8, 48.7)	41.2 (38.8, 43.7)	-5.0 (-7.5, -2.5)	6.6 (5.9, 7.5)	7.2 (6.4, 8.2)	0.6 (-0.2, 1.4)
2 months	31.0 (28.6, 33.5)	35.0 (32.5, 37.7)	4.0 (1.5, 6.6)	55.7 (53.1, 58.2)	50.1 (47.4, 52.7)	-5.6 (-8.3, -2.9)	13.3 (12.0, 14.9)	14.9 (13.3, 16.6)	1.6 (0.0, 3.1)
3 months	21.4 (19.2, 23.7)	24.7 (22.3, 27.3)	3.3 (0.9, 5.7)	59.6 (57.0, 62.1)	53.7 (51.0, 56.4)	-5.8 (-8.5, -3.2)	19.1 (17.2, 21.1)	21.6 (19.5, 23.8)	2.5 (0.4, 4.5)
6 months	8.7 (7.3, 10.3)	10.6 (8.9, 12.5)	1.9 (0.2, 3.5)	60.5 (57.9, 63.0)	54.3 (51.5, 57.0)	-6.2 (-8.9, -3.5)	30.8 (28.3, 33.4)	35.2 (32.4, 38.1)	4.4 (1.6, 7.1)
12 months	2.3 (1.7, 3.0)	2.9 (2.2, 3.9)	0.7 (-0.1, 1.4)	51.7 (48.6, 54.7)	44.9 (41.6, 48.1)	-6.8 (-10.0, -3.6)	46.1 (43.0, 49.2)	52.2 (48.8, 55.6)	6.1 (2.8, 9.4)
**Stage missing**									
1 month	49.1 (44.5, 53.7)	48.3 (43.4, 53.2)	-0.8 (-5.9, 4.3)	38.3 (34.2, 42.7)	36.4 (32.0, 41.1)	-1.9 (-6.7, 2.9)	12.6 (10.9, 14.5)	15.2 (13.2, 17.6)	2.7 (0.8, 4.5)
2 months	39.4 (34.7, 44.2)	38.1 (33.3, 43.2)	-1.3 (-6.5, 4.0)	43.3 (38.9, 47.7)	41.0 (36.3, 45.9)	-2.3 (-7.3, 2.7)	17.4 (15.2, 19.8)	20.9 (18.2, 23.8)	3.5 (1.0, 6.0)
3 months	33.5 (28.9, 38.3)	32.0 (27.3, 37.0)	-1.5 (-6.7, 3.6)	45.6 (41.2, 50.1)	43.1 (38.3, 48.0)	-2.5 (-7.5, 2.5)	20.9 (18.3, 23.8)	24.9 (21.9, 28.3)	4.0 (1.1, 6.9)
6 months	23.5 (19.5, 28.1)	21.7 (17.6, 26.4)	-1.8 (-6.5, 2.8)	47.8 (43.3, 52.4)	44.9 (40.1, 49.8)	-2.9 (-8.0, 2.1)	28.6 (25.3, 32.3)	33.4 (29.5, 37.6)	4.8 (1.0, 8.6)
12 months	14.6 (11.4, 18.5)	12.7 (9.6, 16.5)	-1.9 (-5.6, 1.8)	46.6 (41.7, 51.5)	43.3 (38.2, 48.6)	-3.2 (-8.6, 2.2)	38.9 (34.4, 43.6)	44.0 (39.0, 49.1)	5.1 (0.1, 10.1)

These results are also shown in Figure 2 and 3. These are fully conditional estimates where socioeconomic status was set at 1st (least deprived) and 5th (most deprived) quintile, age at 75 years old, all other covariates were at their corresponding reference groups (i.e., men, white, no heart failure, no myocardial infarction, no diabetes with complications, no chronic pulmonary disease, and standard referral).

Table S7. Length of staying alive and untreated, and alive and untreated, and days of life lost in patients with colon or rectal cancer by stage in England between 2012 and 2016

Follow-up, days	Length of staying alive and untreated, days			Length of staying alive and treated, days			Days of life lost, days		
	Least deprived	Most deprived	Difference	Least deprived	Most deprived	Difference	Least deprived	Most deprived	Difference
**Colon**									
**Stage I**									
1 month	16.7 (16.2, 17.3)	17.2 (16.6, 17.8)	0.5 (-0.1, 1.1)	12.9 (12.4, 13.4)	12.4 (11.8, 13.0)	-0.5 (-1.1, 0.0)	0.4 (0.3, 0.4)	0.4 (0.3, 0.4)	0.0 (-0.0, 0.1)
2 months	26.7 (25.6, 27.9)	27.7 (26.5, 29.0)	1.0 (-0.2, 2.3)	32.2 (31.1, 33.3)	31.0 (29.8, 32.3)	-1.2 (-2.4, 0.0)	1.1 (1.0, 1.2)	1.2 (1.1, 1.4)	0.1 (0.0, 0.3)
3 months	33.9 (32.3, 35.6)	35.4 (33.5, 37.4)	1.5 (-0.4, 3.4)	54.0 (52.4, 55.6)	52.1 (50.3, 54.0)	-1.8 (-3.7, -0.0)	2.1 (1.9, 2.4)	2.5 (2.1, 2.8)	0.3 (0.1, 0.6)
6 months	47.3 (44.3, 50.5)	49.9 (46.5, 53.6)	2.6 (-0.9, 6.1)	126.1 (123.2, 129.0)	122.2 (118.8, 125.6)	-3.9 (-7.3, -0.6)	6.6 (5.9, 7.4)	7.9 (6.9, 9.1)	1.3 (0.3, 2.3)
12 months	59.3 (54.7, 64.4)	63.1 (57.7, 69.1)	3.8 (-1.8, 9.3)	280.4 (275.6, 285.2)	271.9 (265.9, 278.0)	-8.5 (-14.2, -2.7)	20.3 (18.0, 22.9)	25.0 (21.6, 28.9)	4.7 (1.4, 8.0)
**Stage II**									
1 month	20.3 (20.0, 20.7)	20.8 (20.5, 21.2)	0.5 (0.2, 0.9)	9.3 (9.0, 9.7)	8.8 (8.4, 9.1)	-0.6 (-0.9, -0.2)	0.4 (0.3, 0.4)	0.4 (0.4, 0.5)	0.1 (0.0, 0.1)
2 months	30.1 (29.3, 30.9)	31.4 (30.5, 32.3)	1.3 (0.4, 2.2)	28.8 (28.0, 29.6)	27.2 (26.3, 28.2)	-1.6 (-2.4, -0.7)	1.1 (1.0, 1.3)	1.4 (1.2, 1.6)	0.2 (0.1, 0.4)
3 months	35.9 (34.7, 37.1)	37.9 (36.5, 39.4)	2.0 (0.6, 3.4)	51.9 (50.7, 53.1)	49.3 (47.9, 50.8)	-2.6 (-3.9, -1.2)	2.2 (2.0, 2.5)	2.8 (2.4, 3.1)	0.5 (0.3, 0.8)
6 months	45.7 (43.6, 47.9)	49.3 (46.7, 52.0)	3.6 (1.1, 6.1)	127.4 (125.3, 129.6)	122.0 (119.3, 124.7)	-5.5 (-8.0, -2.9)	6.9 (6.2, 7.7)	8.8 (7.8, 9.9)	1.9 (1.0, 2.8)
12 months	55.1 (51.8, 58.6)	60.7 (56.6, 65.1)	5.6 (1.6, 9.6)	284.7 (280.8, 288.5)	272.8 (267.8, 277.9)	-11.8 (-16.4, -7.3)	20.3 (18.2, 22.6)	26.5 (23.5, 29.9)	6.3 (3.5, 9.0)
**Stage III**									
1 month	21.0 (20.7, 21.3)	22.1 (21.7, 22.4)	1.1 (0.7, 1.4)	8.6 (8.3, 9.0)	7.6 (7.3, 7.9)	-1.1 (-1.4, -0.8)	0.3 (0.3, 0.4)	0.4 (0.3, 0.4)	0.0 (-0.0, 0.0)
2 months	31.1 (30.4, 31.9)	33.9 (33.0, 34.7)	2.8 (1.9, 3.6)	27.7 (27.0, 28.5)	24.8 (24.0, 25.7)	-2.9 (-3.7, -2.1)	1.2 (1.1, 1.3)	1.3 (1.2, 1.4)	0.1 (0.0, 0.2)
3 months	37.1 (35.9, 38.2)	41.3 (40.0, 42.7)	4.3 (3.0, 5.6)	50.6 (49.4, 51.7)	45.9 (44.6, 47.3)	-4.6 (-5.9, -3.4)	2.4 (2.2, 2.6)	2.7 (2.5, 3.0)	0.4 (0.1, 0.6)
6 months	47.0 (45.0, 49.1)	54.7 (52.2, 57.3)	7.7 (5.3, 10.1)	125.0 (122.9, 127.1)	115.6 (113.0, 118.2)	-9.4 (-11.9, -7.0)	8.0 (7.3, 8.8)	9.7 (8.8, 10.8)	1.7 (0.9, 2.6)
12 months	56.6 (53.5, 60.0)	68.6 (64.5, 73.0)	12.0 (8.1, 15.9)	277.0 (273.1, 280.9)	257.8 (252.7, 263.0)	-19.2 (-23.8, -14.5)	26.4 (24.1, 28.9)	33.6 (30.4, 37.1)	7.2 (4.3, 10.1)
**Stage IV**									
1 month	19.7 (19.3, 20.2)	21.1 (20.7, 21.5)	1.3 (0.9, 1.7)	8.9 (8.5, 9.3)	7.4 (7.0, 7.8)	-1.5 (-1.9, -1.1)	1.4 (1.3, 1.5)	1.5 (1.4, 1.7)	0.2 (0.1, 0.3)
2 months	33.3 (32.4, 34.2)	35.9 (35.0, 36.9)	2.7 (1.7, 3.6)	21.0 (20.0, 21.9)	17.6 (16.7, 18.5)	-3.4 (-4.3, -2.5)	5.8 (5.4, 6.2)	6.5 (6.0, 7.0)	0.7 (0.3, 1.1)
3 months	42.4 (41.0, 43.8)	46.2 (44.8, 47.7)	3.9 (2.4, 5.3)	34.9 (33.5, 36.5)	29.4 (28.0, 31.0)	-5.5 (-7.0, -4.0)	12.7 (11.9, 13.5)	14.3 (13.4, 15.3)	1.7 (0.8, 2.5)
6 months	56.0 (53.5, 58.6)	62.5 (59.7, 65.4)	6.5 (3.8, 9.2)	81.2 (78.1, 84.3)	69.0 (65.8, 72.3)	-12.2 (-15.4, -9.0)	42.8 (40.5, 45.3)	48.6 (45.9, 51.4)	5.7 (3.2, 8.3)
12 months	63.1 (59.7, 66.7)	72.0 (68.0, 76.3)	9.0 (5.0, 12.9)	169.3 (163.0, 175.8)	144.7 (138.0, 151.8)	-24.6 (-31.4, -17.8)	127.6 (121.9, 133.7)	143.2 (136.7, 150.1)	15.6 (9.2, 22.0)
**Stage missing**									
1 month	19.5 (18.6, 20.4)	20.7 (19.9, 21.6)	1.3 (0.4, 2.2)	7.3 (6.6, 8.1)	5.8 (5.1, 6.6)	-1.5 (-2.3, -0.7)	3.2 (2.9, 3.5)	3.5 (3.1, 3.8)	0.2 (-0.0, 0.5)
2 months	34.4 (32.5, 36.3)	37.0 (35.2, 39.0)	2.7 (0.7, 4.6)	16.8 (15.2, 18.6)	13.5 (11.9, 15.3)	-3.4 (-5.2, -1.5)	8.8 (8.1, 9.6)	9.5 (8.7, 10.4)	0.7 (-0.0, 1.4)
3 months	46.9 (44.0, 49.9)	50.8 (47.9, 53.9)	4.0 (0.9, 7.1)	27.0 (24.4, 29.9)	21.7 (19.2, 24.6)	-5.3 (-8.2, -2.5)	16.1 (14.8, 17.5)	17.4 (16.0, 19.1)	1.4 (0.0, 2.7)
6 months	76.7 (70.9, 83.0)	84.1 (78.1, 90.6)	7.5 (1.1, 13.8)	59.0 (53.5, 65.0)	47.7 (42.2, 53.8)	-11.3 (-17.4, -5.2)	44.4 (40.9, 48.1)	48.2 (44.4, 52.4)	3.9 (0.3, 7.4)
12 months	117.7 (106.7, 129.9)	130.6 (118.9, 143.4)	12.9 (0.6, 25.2)	122.0 (110.5, 134.6)	99.0 (87.5, 112.1)	-22.9 (-35.6, -10.2)	120.3 (111.5, 129.8)	130.4 (120.6, 140.8)	10.0 (0.6, 19.4)

Follow-up, days	Length of staying alive and untreated, days			Length of staying alive and treated, days			Days of life lost, days		
	Least deprived	Most deprived	Difference	Least deprived	Most deprived	Difference	Least deprived	Most deprived	Difference
**Rectal**									
**Stage I**									
1 month	19.6 (19.1, 20.2)	19.9 (19.3, 20.5)	0.3 (-0.3, 0.8)	10.0 (9.5, 10.5)	9.7 (9.2, 10.3)	-0.3 (-0.8, 0.3)	0.3 (0.3, 0.4)	0.4 (0.3, 0.4)	0.0 (-0.0, 0.0)
2 months	31.7 (30.6, 32.9)	32.3 (31.0, 33.7)	0.6 (-0.7, 1.9)	27.2 (26.1, 28.4)	26.6 (25.3, 27.9)	-0.7 (-1.9, 0.6)	1.0 (0.9, 1.1)	1.1 (1.0, 1.3)	0.1 (-0.0, 0.2)
3 months	39.9 (38.1, 41.7)	40.8 (38.8, 42.9)	0.9 (-1.1, 2.9)	48.2 (46.5, 49.9)	47.0 (45.1, 49.0)	-1.1 (-3.1, 0.8)	1.9 (1.7, 2.2)	2.2 (1.9, 2.5)	0.3 (-0.0, 0.5)
6 months	52.5 (49.4, 55.8)	54.1 (50.4, 58.0)	1.6 (-2.2, 5.3)	121.5 (118.5, 124.6)	118.8 (115.2, 122.4)	-2.8 (-6.3, 0.8)	6.0 (5.2, 6.8)	7.2 (6.1, 8.4)	1.2 (0.2, 2.2)
12 months	59.3 (55.0, 64.0)	61.5 (56.4, 67.0)	2.1 (-3.2, 7.4)	282.2 (277.6, 286.8)	275.2 (269.4, 281.1)	-7.0 (-12.7, -1.4)	18.5 (16.1, 21.2)	23.3 (19.7, 27.6)	4.9 (1.3, 8.4)
**Stage II**									
1 month	24.8 (24.4, 25.1)	25.0 (24.6, 25.4)	0.3 (-0.1, 0.6)	5.1 (4.7, 5.4)	4.8 (4.4, 5.1)	-0.3 (-0.6, 0.0)	0.2 (0.1, 0.2)	0.2 (0.2, 0.3)	0.0 (0.0, 0.1)
2 months	38.3 (37.2, 39.4)	39.1 (38.0, 40.4)	0.9 (-0.3, 2.0)	21.1 (20.0, 22.2)	20.0 (18.8, 21.2)	-1.1 (-2.2, 0.0)	0.7 (0.5, 0.8)	0.9 (0.7, 1.1)	0.2 (0.0, 0.4)
3 months	45.4 (43.6, 47.2)	46.9 (44.9, 48.9)	1.5 (-0.5, 3.4)	43.1 (41.4, 44.9)	41.2 (39.2, 43.2)	-2.0 (-3.9, -0.1)	1.5 (1.2, 1.8)	2.0 (1.6, 2.5)	0.5 (0.1, 0.9)
6 months	56.6 (53.3, 60.1)	59.3 (55.5, 63.3)	2.7 (-1.1, 6.5)	118.1 (114.7, 121.5)	113.3 (109.4, 117.4)	-4.7 (-8.6, -0.9)	5.4 (4.4, 6.5)	7.4 (6.0, 9.1)	2.0 (0.7, 3.4)
12 months	68.9 (63.4, 74.9)	72.8 (66.4, 79.8)	3.9 (-2.5, 10.3)	272.5 (266.1, 279.1)	261.4 (253.5, 269.5)	-11.1 (-18.6, -3.6)	18.6 (15.3, 22.6)	25.8 (21.1, 31.7)	7.2 (2.5, 11.9)
**Stage III**									
1 month	26.0 (25.8, 26.2)	26.2 (26.0, 26.4)	0.2 (0.1, 0.4)	3.9 (3.7, 4.1)	3.7 (3.5, 3.9)	-0.3 (-0.4, -0.1)	0.1 (0.1, 0.1)	0.1 (0.1, 0.1)	0.0 (-0.0, 0.0)
2 months	40.2 (39.5, 40.9)	41.1 (40.4, 41.9)	1.0 (0.3, 1.7)	19.4 (18.7, 20.1)	18.4 (17.7, 19.1)	-1.0 (-1.7, -0.3)	0.4 (0.3, 0.5)	0.5 (0.4, 0.6)	0.1 (0.0, 0.2)
3 months	46.8 (45.6, 48.0)	48.4 (47.2, 49.7)	1.6 (0.4, 2.9)	42.2 (41.1, 43.4)	40.3 (39.1, 41.6)	-1.9 (-3.1, -0.7)	1.0 (0.8, 1.2)	1.2 (1.0, 1.5)	0.3 (0.0, 0.5)
6 months	55.6 (53.5, 57.7)	58.5 (56.2, 60.9)	2.9 (0.6, 5.2)	119.9 (117.7, 122.1)	115.7 (113.3, 118.2)	-4.2 (-6.6, -1.8)	4.5 (3.8, 5.3)	5.8 (4.9, 6.9)	1.3 (0.3, 2.2)
12 months	64.7 (61.3, 68.3)	68.8 (64.9, 72.8)	4.1 (0.2, 7.9)	276.7 (272.4, 280.9)	267.5 (262.5, 272.6)	-9.1 (-14.0, -4.3)	18.6 (16.0, 21.7)	23.7 (20.3, 27.7)	5.1 (1.5, 8.6)
**Stage IV**									
1 month	18.9 (18.3, 19.6)	20.0 (19.4, 20.6)	1.1 (0.5, 1.7)	10.1 (9.5, 10.7)	8.9 (8.3, 9.5)	-1.2 (-1.8, -0.6)	1.0 (0.9, 1.1)	1.1 (0.9, 1.2)	0.1 (-0.0, 0.2)
2 months	30.4 (29.1, 31.8)	32.8 (31.5, 34.2)	2.4 (1.0, 3.8)	25.6 (24.3, 27.0)	22.8 (21.5, 24.2)	-2.8 (-4.2, -1.4)	4.0 (3.6, 4.5)	4.4 (3.9, 5.0)	0.4 (-0.1, 0.9)
3 months	38.2 (36.2, 40.2)	41.7 (39.6, 43.8)	3.5 (1.4, 5.6)	42.9 (40.9, 45.1)	38.4 (36.4, 40.7)	-4.5 (-6.7, -2.3)	8.9 (8.0, 9.9)	9.9 (8.9, 11.1)	1.0 (-0.0, 2.0)
6 months	50.6 (47.1, 54.3)	56.3 (52.5, 60.4)	5.8 (1.9, 9.7)	97.7 (93.5, 102.0)	87.7 (83.3, 92.3)	-9.9 (-14.4, -5.4)	31.8 (28.9, 34.9)	35.9 (32.7, 39.5)	4.2 (1.0, 7.4)
12 months	58.9 (53.8, 64.5)	66.8 (61.0, 73.1)	7.9 (2.0, 13.7)	199.3 (190.8, 208.2)	177.6 (168.5, 187.2)	-21.7 (-31.0, -12.4)	101.8 (94.2, 110.0)	115.6 (107.2, 124.8)	13.8 (5.3, 22.4)
**Stage missing**									
1 month	18.1 (16.9, 19.4)	18.0 (16.7, 19.3)	-0.1 (-1.4, 1.2)	9.3 (8.3, 10.5)	8.9 (7.8, 10.2)	-0.4 (-1.7, 0.8)	2.6 (2.2, 3.0)	3.1 (2.7, 3.6)	0.5 (0.2, 0.9)
2 months	31.2 (28.7, 34.0)	30.8 (28.1, 33.7)	-0.4 (-3.3, 2.5)	21.7 (19.4, 24.2)	20.6 (18.2, 23.4)	-1.1 (-3.8, 1.7)	7.1 (6.2, 8.2)	8.6 (7.5, 9.9)	1.5 (0.4, 2.5)
3 months	42.1 (38.2, 46.4)	41.3 (37.2, 45.8)	-0.8 (-5.3, 3.6)	35.0 (31.5, 39.0)	33.3 (29.5, 37.5)	-1.8 (-6.0, 2.4)	12.9 (11.2, 14.7)	15.5 (13.5, 17.7)	2.6 (0.8, 4.5)
6 months	67.2 (59.6, 75.8)	64.8 (56.9, 73.8)	-2.4 (-11.2, 6.5)	77.4 (70.1, 85.5)	73.1 (65.3, 82.0)	-4.3 (-13.0, 4.4)	35.4 (31.1, 40.2)	42.0 (37.0, 47.8)	6.6 (1.8, 11.5)
12 months	100.3 (86.3, 116.6)	94.5 (80.4, 111.1)	-5.8 (-22.1, 10.5)	162.9 (147.9, 179.4)	153.0 (136.9, 171.0)	-9.9 (-27.5, 7.8)	96.8 (85.7, 109.3)	112.5 (99.9, 126.7)	15.7 (3.0, 28.4)

These results are also shown in Figure 4. These are fully conditional estimates where socioeconomic status was set at 1st (least deprived) and 5th (most deprived) quintile, age at 75 years old, all other covariates were at their corresponding reference groups (i.e., men, white, no heart failure, no myocardial infarction, no diabetes with complications, no chronic pulmonary disease, and standard referral).
